# Transcriptomic Analysis Provides Insights to Reveal the *bmp6* Function Related to the Development of Intermuscular Bones in Zebrafish

**DOI:** 10.3389/fcell.2022.821471

**Published:** 2022-05-12

**Authors:** Huan Xu, Guangxiang Tong, Ting Yan, Le Dong, Xiaoxing Yang, Dongyu Dou, Zhipeng Sun, Tianqi Liu, Xianhu Zheng, Jian Yang, Xiaowen Sun, Yi Zhou, Youyi Kuang

**Affiliations:** ^1^ Heilongjiang River Fisheries Research Institute of Chinese Academy of Fishery Sciences, Harbin, China; ^2^ National and Local Joint Engineering Laboratory for Freshwater Fish Breeding, Harbin, China; ^3^ Key Laboratory of Freshwater Aquatic Biotechnology and Breeding, Ministry of Agriculture and Rural Affairs, Harbin, China; ^4^ Heilongjiang Provincial Key Laboratory of Hard Tissue Development and Regeneration, The Second Affiliated Hospital of Harbin Medical University, Harbin, China; ^5^ College of Fisheries and Life Science, Shanghai Ocean University, Shanghai, China; ^6^ Institute of Mariculture Breeding and Seed Industry, Zhejiang Wanli University, Ningbo, China; ^7^ Stem Cell Program of Boston Children’s Hospital, Division of Hematology/Oncology, Boston Children’s Hospital and Dana Farber Cancer Institute, Harvard Medical School, Boston, MA, United States

**Keywords:** intermuscular bones, zebrafish, BMP6, bone development, SIK1

## Abstract

Intermuscular bones (IBs) are small, hard-boned spicules located in the muscle tissue that mainly exist in the myosepta of lower teleosts, which hurt the edibleness and economic value of fish. The study of the development of IBs is very important for freshwater aquaculture fish, but the molecular mechanism of its formation and the key regulatory genes remain unclear. In this study, we first constructed two types of zebrafish mutants (the mutants losing IBs and the mutants with partial deletion of IBs) by knocking out *bmp6*. We then carried out a transcriptomic analysis to reveal the role of *bmp6* in the developmental mechanism of IBs; we used the caudal musculoskeletal tissues of these mutants and wild-type zebrafish at three development stages (20, 45, and 60 dph) to perform transcriptomic analysis. The results showed that the deficiency of *bmp6* upregulated *sik1* and activated the TNF-A signaling *via* the NF-KB pathway, which inhibited the development of osteoblasts and promoted osteoclast formation, thereby inhibiting the formation of IBs. These results provided insights to understand the role of *bmp6* in the development of IBs in zebrafish and are useful for selective breeding of IBs in cyprinids.

## Introduction

As one of the most sustainable sources of protein for humans, aquaculture plays an important role in agricultural production (Nie C. H. et al., 2019). Most of the freshwater aquaculture fishes, especially Cypriniformes species, possessed a certain amount of intermuscular bones (IBs) (Wan et al., 2016). IBs are small, hard-boned spicules located in the muscle tissue on both sides of the vertebrae; they mainly exist in the myosepta of lower teleosts (Li et al., 2013; Nie C.-H. et al., 2019). IBs are difficult to remove, so they may be dangerous when consumed and are not conducive to the processing of aquatic products (Li et al., 2021). Therefore, IBs have been recognized as a noteworthy feature in aquaculture breeding programs because of their negative effect on the edibleness and economic value of fishes (Nie C. H. et al., 2019).

It is widely accepted that IBs arise undergoing intramembranous ossification of tendons in the myoseptum, in which mesenchymal cells directly differentiate into osteoblasts and begin to ossify (Nie C.-H. et al., 2019). Omics studies showed that IB development involved many pathways, such as Wnt pathway, TGF-β pathway, MAPK, calcium signaling, thyroid hormone signaling, hedgehog signaling, TNF signaling, NF-kappa B pathway, and osteoclast differentiation(Li et al., 2021). Genes that may influence IB development include *sxca*, *msxC*, *sost*, and *bmps* (bone morphogenetic proteins). *MsxC* may induce epithelial–mesenchymal interactions during the formation of IBs (Lv et al., 2015), and *sost* inhibits the Wnt and BMP signaling pathways to regulate bone development (Qin et al., 2013). Transcriptomic analyses in blunt snout bream at four developmental stages (S1–S4) of IBs (Nie C.-H. et al., 2019) showed that *bmp3*, *bmp4*, *bmp5*, and *bmp8a* increase significantly during S2 (several small IBs started to ossify in the tail part) and *bmp7b* and *bmp16* are highly expressed during S3 (IBs appeared rapidly), suggesting that they may participate in osteoblast differentiation and IB maturation (Zhang et al., 2018; Nie C.-H. et al., 2019). Zebrafish *scxa* ^*−/−*^ mutants showed significant reductions in the total number of IBs (Nie et al., 2020); however, *scxa* ^*−/−*^ mutants presented some negative effects such as cranial tendon and ligament defects and deficient ribs (Kague et al., 2019). MicroRNAs (miRNAs) are small non-coding RNAs comprising about 22 nucleotides and target mRNAs and control their degradation (Seco-Cervera et al., 2018). MiRNAs are known to be involved in a variety of physiological processes, and previous studies showed that some miRNAs such as let-7d-3p and miR199-3p had significantly higher expression in IBs in blunt snout bream (Wan et al., 2015). Although previous studies investigated some genes and pathways involved in the development of IBs, the molecular mechanism of IB formation has not been fully studied, and the key regulatory genes related to IB development without affecting other systematic functions remain unclear.

In our previous study, we performed a QTL analysis on the number of IBs in *Cyprinus carpio* and found a suggestive QTL (90% chromosome-wide threshold) containing the *bmp6* gene (Tang et al., 2020). *Bmp6* is related to osteoblast and bone development (Chen et al., 2012; Zhu et al., 2012), and BMP signaling is required for tendon ossification (Dai et al., 2020; Huang et al., 2021); therefore, we speculated that *bmp6* may be involved with the development of IBs. In this study, we first knocked out *bmp6* through CRISPR/Cas9 and obtained two alleles of IB-deficient mutants with normal growth (the mutants completely lost IBs and the mutants with partial deletion of IBs), which suggested that *bmp6* may be a key regulatory gene in IB formation. In order to study the role of *bmp6* in IB development, we then performed transcriptomic analyses on the two alleles of mutants and wild-type zebrafish at 20 45 and 60 dph (days post hatched) stages, respectively. The results obtained in our study will be valuable for studies on the molecular mechanism of IB formation in zebrafish and provide useful tools for breeding IB-lacked varieties by gene-editing technology in cyprinids.

## Materials and Methods

### Zebrafish Maintenance

Zebrafish (AB strain) were bought from the China Zebrafish Resource Center (CZRC, http://www.zfish.cn) and maintained by the aquatic facility at Heilongjiang River Fisheries Research Institute of Chinese Academy of Fishery Sciences (HRFRI) according to the guidance of the zebrafish book (Westerfield, 2007). For micro-injection, zebrafish were mated with one male and one female per tank. After spawning, the embryos were collected and washed twice using embryo water and then incubated at 28.5°C, and the live embryos were transferred to fresh petri dishes every day. The larvae were fed four times per day with paramecium.

### RNA Fluorescence *In Situ* Hybridization

To detect the spatial expression in caudal musculoskeletal tissue, we used Tg (Ola.sp7-GFP) zebrafish from CZRC to carry out cryosectioning and RNA-FISH. RNA-FISH was carried out with the SABER-FISH protocol proposed by Kishi et al. (2018); Kishi et al. (2019) used 90-dph samples. The probe sequence was extracted from the zebrafish probe sequence library designed by Beliveau et al. (2018) (https://oligopaints.hms.harvard.edu/genome-files). The mRNA of *bmp6*, *sik1*, and *acp5b* was detected with Alexa Fluor 647 fluorescent probes, while mRNA of *scxa*, *tnmd*, and *xirp2a* was detected with Alexa Fluro 532 probes, and the nucleus was stained with DAPI dye (Thermo Fisher, CA, United States). Sequences of probes used for *bmp6, sik1, acp5b, scxa, tnmd*, and *xirp2a* RNA-FISH are shown as in Supplementary Table S1. The images were captured by a Nikon Eclipse Ti inverted fluorescence microscope (Nikon, Shanghai) and were processed by ImageJ.

### *Bmp6* Knockout

**FIGURE 1:**
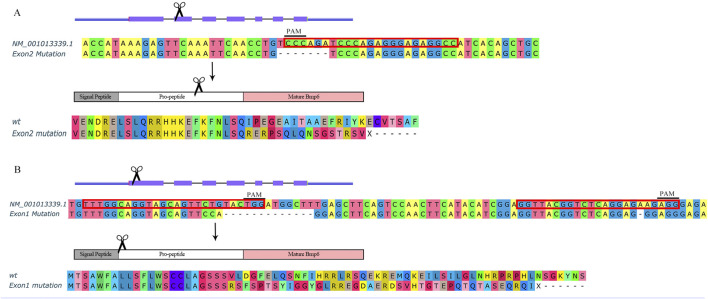
Schematic of *bmp6* knockout. **(A)** Knockout *bmp6* on exon 2 in group M. **(B)** Knockout *bmp6* on exon 1 in group A. Sequences with red border are target sites including PAM sites for CRISPR/Cas9.

The *bmp6* knockout targets for CRISPR/Cas9 were designed with CHOPCHOP service (Labun et al., 2019). The sgRNAs were synthesized by using the HiScribe T7 Quick High Yield RNA Synthesis Kit (E2050, NEB, MA, United States). After mixing 5 μM sgRNAs and 5 μM Cas9 protein (M0646, NEB, MA, United States), 1 nl mixture was micro-injected to the yolk of embryos at the single-cell stage. The *bmp6* gene in zebrafish has seven exons; schematic of the *bmp6* gene structure and the knockout targets and mutational sequences are shown in Figure 1.

### Bone Staining and Micro-CT

The mutants and their sibling wild-type zebrafish were stained with Alizarin red and Alcian blue according to the method described by Yang et al. (2020), and images were taken by an Olympus BX53 fluorescent microscope. Fish at 60 dph were used to observe the ossified skeleton by staining with Alizarin red, and fish at 6 dph were used to observe the skull structure by staining with Alcian blue. Furthermore, the phenotypes of mutants and wild-type zebrafish at 90 dph were verified using the Quantum GX micro-CT Imaging System (PerkinElmer, MA, United States) with 70 kV, 88 μA and 14 min scan time.

### Fish for Transcriptome Analysis

**FIGURE 2:**
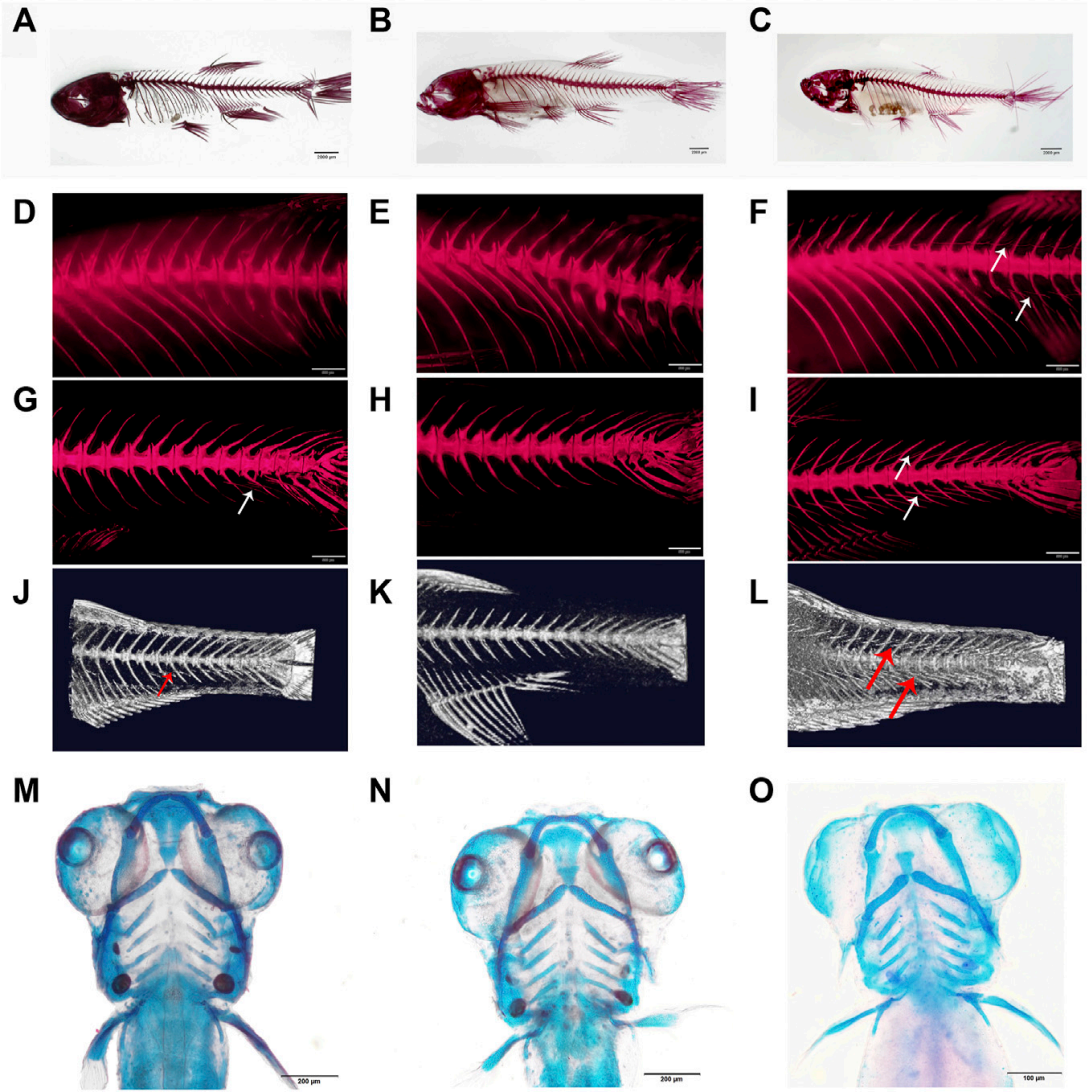
Phenotypes of *bmp6* knockout mutants. **(A–C)** Bone staining with Alizarin red of M mutant, that is, IB partially deficient mutant (A), A mutant, that is, IB depletion mutant (B) and wild-type zebrafish (C) at 60 dph. **(D–F)** Bone staining of the dorsal part of M mutant, A mutant, and wild-type zebrafish, respectively. **(G–I)** Bone staining of the caudal part of M mutant, A mutant, and wild-type zebrafish, respectively. **(J–L)** Micro-CT images of the caudal skeleton of M mutant, A mutant, and wild-type zebrafish, respectively; the IBs were only found in the ventral part of the M mutant (A,D,G,J), whereas the IBs were lacking in the A mutant (B,E,H,K). **(M–O)** Bone staining of the skull of wild M mutant (M), A mutant (N), and wild-type zebrafish (O) at 6 dph.

Fish used for transcriptome analysis were cultured under similar conditions, including the same size of tanks (10L/tank), the same density (30 fish/tank), and similar water conditions and feeding. We used wild-type zebrafish (named WT, Figure 2C) and two alleles of mutated zebrafish to carry out the transcriptome analysis: the mutants with partial deletion of IBs by knocking out *bmp6* on exon 2 (named M, Figures 1A, 2A) and the mutants lost IBs by knocking out *bmp6* on exon 1 (named A, Figures 1B, 2B). After euthanization with MS222 solution, the skin was removed and the caudal musculoskeletal tissues (between the posterior of anal fin and the anterior of caudal fin) were collected from fish at 20, 45, and 60 dph stages. According to our previous studies (Yang et al., 2019), the IBs in zebrafish started to ossify at 33 dph and almost completely formed at 56 dph, so we choose three stages that were mentioned earlier before ossification, during ossification, and after ossification. Because the RNA from a single fish at 20 dph was not enough to construct mRNA- and miRNA-sequencing libraries, we mixed 5–6 fish as a pool to perform mRNA and miRNA sequencing. The same sample preparation method was conducted for fish at 45 and 60 dph. In addition, we only collected muscles from fish at 60 dph (named 60M dph, schematic of sample separation is shown as Supplementary Figure S1) for discovering differences in the muscle. In total, 37 samples in 12 groups were prepared with 3–4 biological replicates for each group, and the biological replicates of each group are shown in Supplementary Tables S2, S3.

### Library Preparation for mRNA and miRNA Sequencing

Total RNA was extracted using the Mid RNA Extraction Kit (Qiagen, CA, United States) by following the manufacturer’s protocol. The quality of total RNA was measured with Nanodrop 8,000 (Thermo Fisher, MA, United States), agarose gel electrophoresis, and Agilent 2,100 Bioanalyzer (Agilent, CA, United States); the quantity of total RNA was measured using the Qubit 3 Kit (Qiagen, CA, United States). The cDNA-sequencing library was constructed using the Illumina TrueSeq RNA Sample Preparation Kit (Illumina, CA, United States). The miRNA-sequencing library was constructed using the Illumina TrueSeq miRNA Sample Preparation Kit (Illumina, CA, United States). The cDNA libraries were sequenced with 150 pair-end modes using the Illumina Nova6000 platform, while the miRNA libraries were sequenced with the Illumina NextSeq CN500 platform. All library constructions and sequencing were carried out at Berry Genomics Inc, CN.

### Transcriptome Assembly and Quantification

Quality control was determined by Trimmomatic (Bolger et al., 2014) to cut the adapter and bases of the start and end of a read with a quality value less than 30 and to drop the read if it is shorter than 50bp. Read mapping, transcript assembly, and quantification were conducted using Hisat2 and StringTie software according to the protocol described by Pertea et al. (2016) and simply described as follows. The final clean reads were mapped to the *Danio rerio* reference genome (GRCz11) using Hisat2 (version 2.2.1) aligner for mRNA with default parameters. BAM files were sorted by SAMtools (Danecek et al., 2021). We used reference annotation from Ensembl (version 101) to guide the transcript assembly of each sample with StringTie program (version 2.1.4). Then, the output GTF files were merged into a single unified transcript using StringTie merge function. The raw count for each gene was also calculated by using StringTie.

### MicroRNA Identification and Quantification

Quality control was determined by Trimmomatic to cut the adapter and bases of the start and end of a read with a quality value less than 30 and to drop the read if it is shorter than 18 bp. To identify potential miRNAs, clean reads which were longer than 18 bp and shorter than 25 bp were retained. MirPro (Fu and Dong, 2018), a pipeline that integrated a set of published tools including Novoalign, RNAfold, HTseq, and mirDeep2, was used to perform miRNA-sequencing data analysis. We used this pipeline to carry out genome mapping, known miRNA annotation, novel miRNA identification, and miRNA quantification. Mature miRNA sequences and hairpin sequences of zebrafish were downloaded from miRBase (Kozomara et al., 2019). miRanda and TargetScan were used to predict target genes of known miRNAs and novel miRNAs. Parameters for miRanda were -sc 140 -en 1, and TargetScan was carried out with default parameters.

### Differential Expression Analysis of mRNA and miRNA

The raw read counts of each gene were used to represent the expression levels of mRNAs in each sample. Differential expression analyses for mRNA and miRNA between three groups were performed using the Limma (Ritchie et al., 2015) package. The trimmed mean of M values (TMM) (Robinson and Oshlack, 2010) method was used to normalize the sequence libraries, and an absolute value of log2 (fold change) > 1 and a Benjamini–Hochberg adjusted *p*-value (FDR) < 0.05 were set as the filter criteria for significant differential expression.

### Pathway Enrichment Analysis

The pathway enrichment analysis was carried out using the clusterProfiler (Yu et al., 2012) software package using differentially expressed genes and target genes significantly negatively correlated with differentially expressed miRNAs among groups. We used hallmark gene sets from MSigDB as the target pathways, which provide more refined and concise inputs for gene set enrichment analysis (Liberzon et al., 2015). Kyoto Encyclopedia of Genes and Genomes (KEGG) pathways, WikiPathways, and Gene Ontology resource (GO) from MSigDB were also used as target pathways to conduct pathway enrichment analysis.

### Gene Set Enrichment Analysis

General differential analysis often focuses on comparing gene expression differences between the two groups, which tends to omit some genes that are biologically important but have no significant differential expression. Therefore, we applied GSEA (Subramanian et al., 2005) to find potentially valuable information such as the biological characteristics of genes and relationships between gene regulatory networks. GSEA was carried out according to the protocol described by Reimand et al. (2019).

### Real-Time Quantitative Reverse Transcription Polymerase Chain Reaction

The muscle tissues of A mutants and WT zebrafish at 90 dph were dissected for qRT-PCR, RNA was extracted using the method described earlier and cDNA was synthesized using the Revert Aid™ First-Strand cDNA Synthesis Kit (Thermo Fisher, CA, United States). Quantitative RT-PCR was performed using the QuantStudio 6 Flex Real-Time PCR System (Thermo Fisher, CA, United States), and the total volume of qRT-PCR was set to 10 μl, including 5 μl of 2 × Luna Universal qPCR Master Mix (NEB, MA, United States), 0.5 μl of 10 μmol/L forward and reverse primers, 1 μl of 50 ng/μl cDNA, and 3 μl of nuclease-free water. The amplification procedure was as follows: denaturation at 95°C for 60 s, followed by 40 cycles of denaturation at 95°C for 15 s, and extension at 60°C for 30 s. Primer sequences used for qRT-PCR are shown as in Supplementary Table S1; *actb* was used as the reference gene.

## Results

### RNA Fluorescence *In Situ* Hybridization of *bmp6*

**FIGURE 3:**
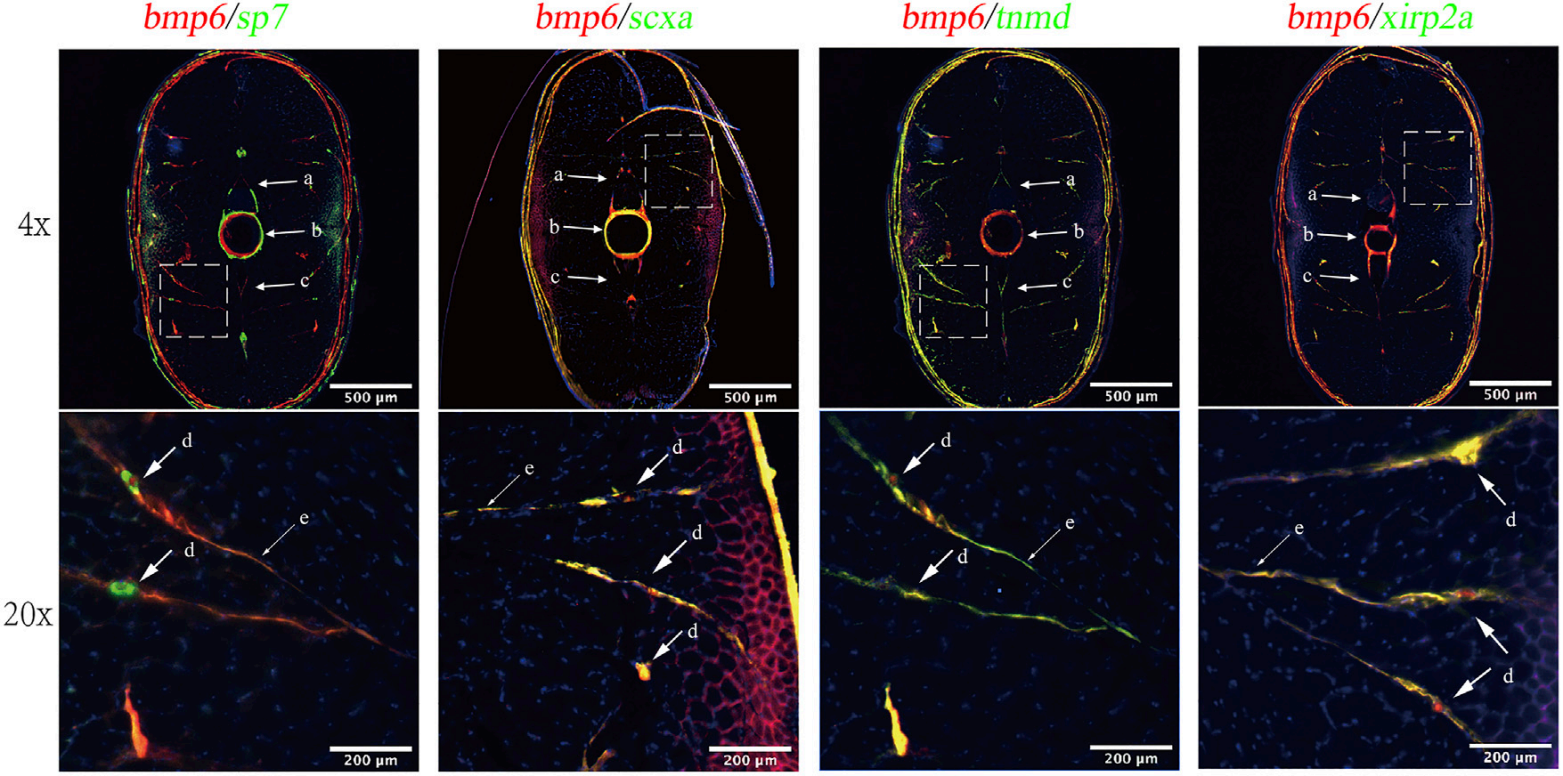
RNA-FISH of bmp6 and co-expression analysis of *sp7, tnmd, scxa, and xirp2a* in the transverse section of zebrafish caudal musculoskeleton tissues. The mRNA of *bmp6* was detected with Alexa Fluor 647 fluorescent probes, while mRNA of *scxa*, *tnmd*, and *xirp2a* was detected with Alexa Fluro 532 probes, and the nucleus was stained with DAPI dye. Intermuscular bones were marked with dotted line boxes at the top of the panel and were enlarged 20 times at the bottom of the panel. Arrows indicated IBs. a: neural arches; b: vertebrae; c: hemal arches; d: IBs; and e: myoseptum.

According to previous studies, IBs arise when undergoing intramembranous ossification in tendon tissue (Nie C.-H. et al., 2019). To analyze the relationship between *bmp6* and IBs, we conducted spatial expression analysis of *bmp6* and co-expression analysis of *sp7*, *tnmd*, *scxa*, and *xirp2a* with *bmp6* to analyze which cells were involved in the development of IBs. The results showed that *bmp6*, *scxa*, *tnmd*, *and xirp2a* were expressed in the myoseptum, whereas *sp7* was only expressed in ossified tendons, that is, IBs. *Bmp6* was also colocalized with *scxa*, *tnmd*, and *xirp2a*, and the expression of *bmp6* and *sp7* was also partially overlapped in IBs and the myoseptum in the transverse section of the caudal tissues (Figure 3, Supplementary Figure S2). These results indicated that *bmp6* was involved in the ossification of tendon in the myoseptum of zebrafish.

### Phenotypes of *bmp6* Knockout

BMPs are growth factors that were first identified as key regulators of bone formation; as a member of the BMP family, Bmp6 affects bone development through the BMP-Smad pathway (Chen et al., 2012); however, Bmp6-deficient mice were viable and fertile (Meynard et al., 2009). In order to investigate whether *bmp6* affects IB development, we designed a target site on exon 2 of *bmp6* (Figure 1A) and two target sites on exon 1 of *bmp6* (Figure 1B). After knocking out at the target site on exon 2 of *bmp6* through CRISPR/Cas9, we obtained the phenotype of IB deficiency in the dorsal part of zebrafish (named M) (Figures 2A,D,G,J, Supplementary Movie S1) compared with the wild-type (named WT) (Figures 2C,F,I,L, Supplementary Movie S2), and the number of IBs in group M (19.8 ± 6.8, n = 10) is significantly decreased (*p* < 0.05) compared with the number of IBs in group WT (91.7 ± 4.6, n = 10). The alignment of the protein sequence inferred by the mutated nucleotide sequence showed that *bmp6* in mutants retained a truncated protein containing about 150 amino acids (Figure 1A); the expression level of *bmp6* in M was significantly (*p* < 0.01) decreased according to our RNA-seq data in this study. We obtained the mutants of complete deficiency of IBs (named A) by knockout of *bmp6* at target sites on exon 1 with CRISPR/Cas9 (Figures 2B,E,H,K, Supplementary Movie S3). Sequence alignment of the mutated protein inferred by nucleotide sequence revealed deletion of Bmp6 protein (Figure 1B), while the expression level of *bmp6* in A did not show a significant difference compared to that of WT. Bone staining of the skull in mutants (M and A) had no significant difference compared with that in WT at 6 dph (Figures 2M–O). There was also no significant difference in bone staining of the head and fin skeletons between the mutants and the wild-type zebrafish at 60 dph (Supplementary Figure S3).

**FIGURE 4:**
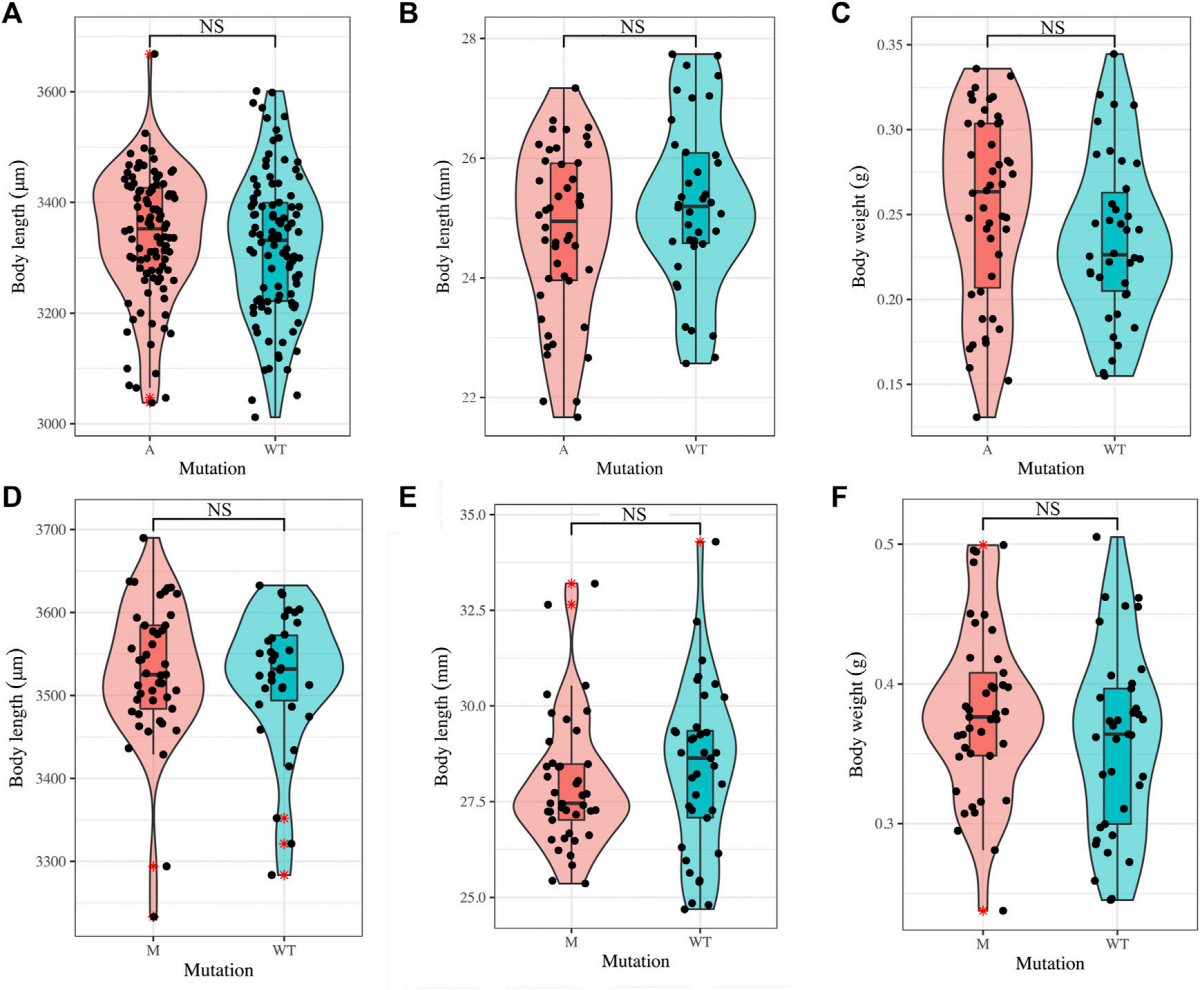
Growth of mutants and wild-type zebrafish. **(A)** Comparison on the larvae body length of wild-type zebrafish (n = 100) and A mutants (n = 100) at 72 hpf. **(B, C)** Comparison on the adult body length (B) and body weight (C) of wild-type zebrafish (n = 38) and A mutants (n = 46) at 90 dph. **(D)** Comparison on the larvae body length of wild-type zebrafish (n = 34) and M mutants (n = 45) at 72 hpf. **(E,F)** Comparison on the adult body length (E) and body weight (F) of wild-type zebrafish (n = 37) and M mutants (n = 41) at 90 dph. Student’s *t*-test was used to test the difference. NS: not significant.

Furthermore, we compared body length in mutants (M and A) and WT at 72 hpf (hours post-fertilized) and 90 dph, and the results of Student’s *t*-test showed that there was no significant difference in the body length between mutants and WT (Figures 4A,B,D,E, Supplementary Table S4), and the comparison of body weight in mutants and WT at 90 dph also showed that no significance occurred (Figures 4C,F, Supplementary Table S4). Our previous studies also showed that the deletion of *bmp6* did not impact skeletal development, muscle development, or the survival of embryos during the embryonic development of zebrafish (Yang et al., 2019; Yang et al., 2020). These results indicated that *bmp6* knockout did not significantly hinder the growth of zebrafish.

### Overview of RNA-Sequencing

We sequenced 37 samples, and quality control was carried out with Trimmomatic and FastQC. The assessment of mRNA-trimmed reads (Supplementary Figures S4A,B) and miRNA-trimmed reads (Supplementary Figures S8C,D) was conducted by FastQC software (version 0.11.9) and summarized by MultiQC (Ewels et al., 2016) (version 1.8). Mean quality scores of mRNA sequences and miRNA sequences were higher than Phred quality 30. These results showed that the sequencing data had high-sequencing quality, and especially mRNA sequences had better uniformity. For mRNA sequencing, 1,907,534,776 raw reads were produced, and 1,792,067,682 clean reads were obtained in total. More than 90% of the clean reads were mapped to the *Danio rerio* reference genome (GRCz11) (Supplementary Table S2), and 22,670 genes were obtained after StringTie processing, and as an example, distribution of junction (Supplementary Figures S5A,B) and assessment of junction saturation (Supplementary Figure S5C) were performed in the B20A2 sample by RSeQC (Wang et al., 2012) software. The number of known junctions and novel junctions reached a plateau which indicated that the current sequencing depth was deep enough to perform differential expression analyses. For miRNA sequencing, 439,200,203 raw reads were produced, and 357,654,864 clean reads were obtained in total. More than 85% of the clean reads were mapped to the *Danio rerio* reference genome (GRCz11) on average (Supplementary Table S3), and identification of miRNAs yielded 374 known miRNAs and 2022 novel miRNAs.

Principal component analysis (PCA) of mRNA expression data (Counts Per Million, CPM) (Robinson et al., 2010) showed that most of the samples from the same period and the same group clustered together; meanwhile, samples from different groups and different stages showed clear separation, especially at 20 dph (Supplementary Figure S6A). Three samples (B60AB8, B60A11, and B60AB11) were not clustered to the corresponding groups and were removed for subsequent analyses. Pearson’s correlation coefficients between the remaining 34 samples showed that samples from the same period and the same group had high correlation (Supplementary Figure S6B). These results indicated that mRNA expression levels between mutants and wild-type and at different development stages were different and samples had good biological repeatability in our study. Since part of miRNA samples were sequenced twice, the Combat method in the SVA (Leek et al., 2019) package was used to remove batch effects for the accuracy of subsequent analysis, and PCA of all samples showed that the same samples from different batches were clustered together after removing the batch effect (Supplementary Figures S6C,D).

### Differential Expression Analysis of mRNA

We investigated the differential expression levels of miRNAs and mRNAs between A, M, and WT groups. Using the significant criteria of log2 (fold change) > 1 and FDR<0.05, we obtained 2,829, 410, and 44 significant differential expression genes (DEGs) in the caudal musculoskeleton between A and WT at 20 dph (20 dph A vs. WT), 45 dph (45 dph A vs. WT), and 60 dph stages (60 dph A vs. WT), respectively (Supplementary Figure S7A, Table 1); 937, 138, and 621 significant DEGs were obtained in the caudal musculoskeleton between M and WT at 20 dph (20 dph M vs. WT), 45 dph (45 dph M vs. WT), and 60 dph stages (60 dph M vs. WT), respectively (Supplementary Figure S7A, Table 1); and 178 and 1,606 DEGs were obtained in the muscle between A and WT (60 Mdph A vs. WT) and M and WT (60 Mdph M vs. WT) in the 60-Mdph group, respectively (Table 1).

**TABLE 1:** Statistics of differentially expressed mRNAs and miRNAs (A: the mutants without intermuscular bones by knocking out *bmp6* on exon 1, M: the mutants with partial deletion of intermuscular bones by knocking out *bmp6* on exon 2, WT: wild-type; the samples of 60 Mdph were the caudal muscle tissues collected from fish at 60 dph, the others were the musculoskeletal tissues from the caudal parts of fish at 20, 45, and 60 dph).

Comparative group	Upregulated mRNAs	Downregulated mRNAs	Upregulated miRNAs	Downregulated miRNAs
20 dph A vs. M	388	546	27	71
20 dph A vs. WT	1,269	1,560	28	187
20 dph M vs. WT	484	453	42	70
45 dph A vs. M	223	381	59	37
45 dph A vs. WT	104	306	60	24
45 dph M vs. WT	58	80	17	3
60 dph A vs. M	171	239	29	16
60 dph A vs. WT	34	10	4	20
60 dph M vs. WT	342	279	6	17
60 Mdph A vs. M	1,049	924	14	24
60 Mdph A vs. WT	96	82	22	29
60 Mdph M vs. WT	748	858	15	21

Among all the differentially expressed genes, si:ch211-213a13.1 and *si:dkey-253d23.4* were upregulated in the A mutated samples compared with that in WT samples at all three stages (Supplementary Figure S7B). Compared with the WT samples, *si:ch211-213a13.1* and *rttn* were upregulated, whereas *zgc:194221* was downregulated in the M-mutated samples at all three stages (Supplementary Figure S7B).

### Pathway Enrichment Analysis of mRNA

**FIGURE 5:**
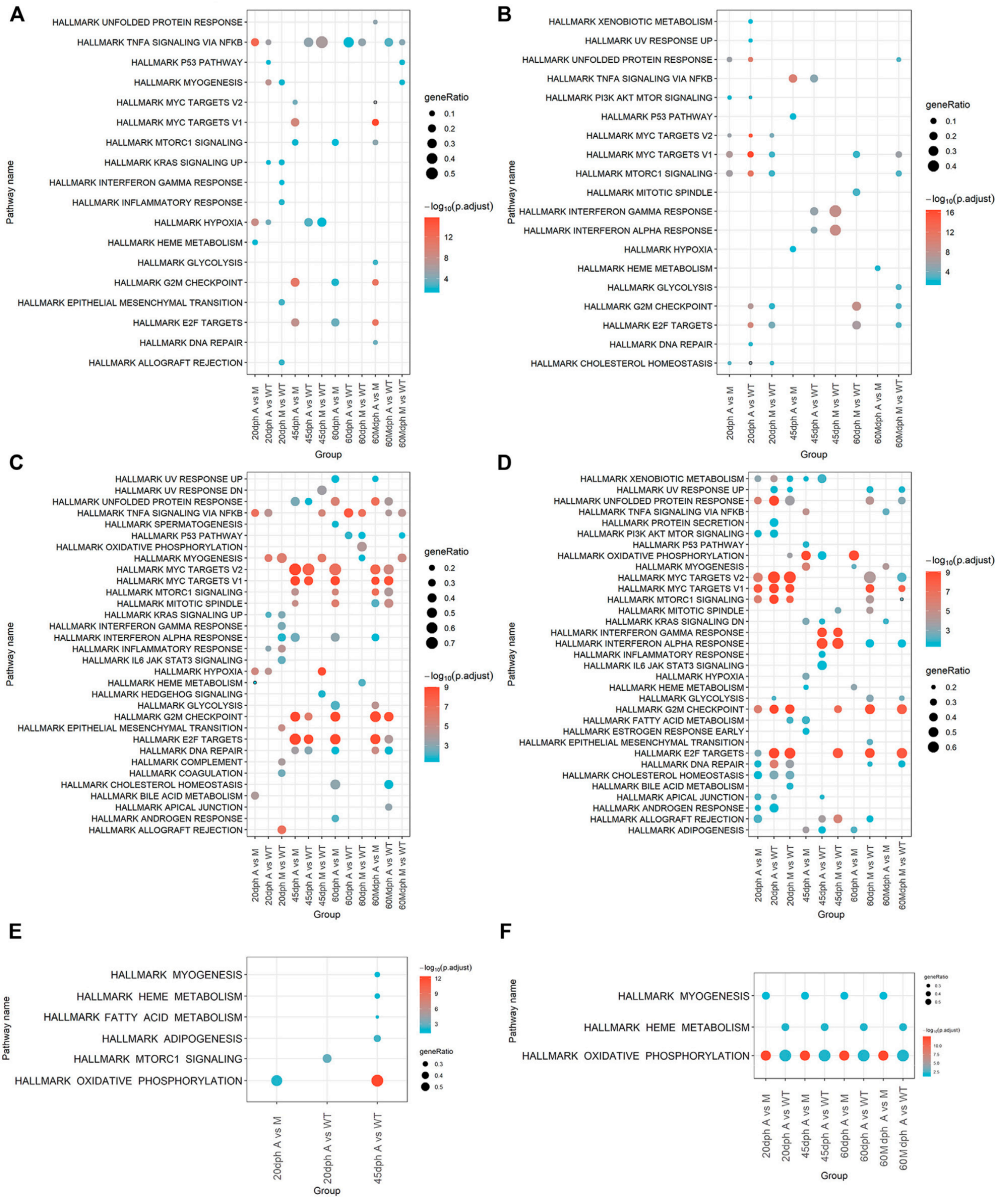
Pathways enriched by DEGs and target genes of DE miRNAs. **(A)** Pathways enriched in upregulated genes between groups. **(B)** Pathways enriched in downregulated DEGs between groups. **(C)** Activated pathways analyzed by GSEA. **(D)** Suppressed pathways analyzed by GSEA. **(E)** Pathways enriched in target genes of upregulated miRNAs between groups. **(F)** Pathways enriched in target genes of downregulated miRNAs between groups. Target genes were predicted by TargetScan and miRanda and had a significantly negative correlation (*p* < 0.05) with DE miRNAs.

The pathway enrichment analysis was conducted using the genes differentially expressed in each comparative group according to upregulation and downregulation (Table 1). Upregulated genes were enriched in 18 pathways, and downregulated genes were enriched in 19 pathways from Hallmark gene sets in total (Figures 5A,B, Supplementary Table S5). The results of pathway analysis using the KEGG database showed that upregulated genes were enriched in 31 KEGG pathways and downregulated genes were enriched in 64 KEGG pathways (Supplementary Figures S8A,B, Supplementary Table S6). Upregulated genes were also enriched in 135 WikiPathways and 734 GO pathways, while downregulated genes were also enriched in 100 WikiPathways and 816 GO pathways (Supplementary Tables S7, S8).

Moreover, pathway enrichment analysis was also carried out with GSEA. GSEA was performed on gene expression data in different comparative groups. A total of 33 pathways were activated, and 34 pathways were suppressed in total using Hallmark gene sets as target gene sets (Figures 5C,D, Supplementary Table S9); 105 KEGG pathways were activated and 157 KEGG pathways were suppressed in total; 189 WikiPathways were activated and 218 WikiPathways were suppressed; 1817 biological processing GO terms were activated and 2065 biological processing GO terms were suppressed. (Supplementary Figures S8C,D, Supplementary Tables S10–S12).

The results of pathway enrichment analysis showed that knockout of *bmp6* affected bone development. Deficiency of *bmp6* inhibited bone development–related pathways and promoted osteoclast differentiation. The pathway enrichment analysis showed that GO bone development and GO regulation of bone mineralization were suppressed in group M when compared with those in group WT at 60 dph (60 dph M vs. WT) and GO osteoclast differentiation was activated in group M compared with group WT at 20 dph (20 dph M vs. WT) (Supplementary Figure S8E). WP bone morphogenic protein BMP signaling and regulation were also suppressed in group A compared with those in group WT at 45 dph (45 dph A vs. WT) (Supplementary Table S11). WP Type I collagen synthesis in the context of osteogenesis imperfecta was suppressed in group M compared with that in group WT at 60 dph (60 dph M vs. WT) (Supplementary Table S11). WP autosomal recessive osteopetrosis pathways were suppressed in the 60-Mdph A group compared with the 60-Mdph M group (Supplementary Table S11). Wnt signaling pathways regulated the development of osteoblast and bone formation; this pathway was also suppressed in mutant A (20 dph A vs. WT and 60 dph A vs. WT) and mutant M (20 dph M vs. WT and 60 dph M vs. WT) groups at 20 dph and 60 dph. (Supplementary Tables S6, S8, S10–S12).

**FIGURE 6:**
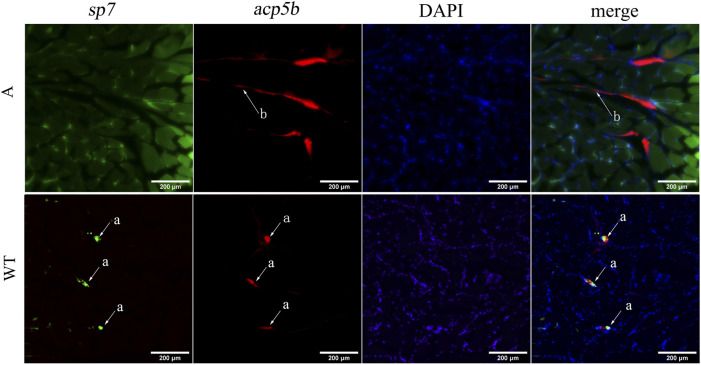
RNA-FISH of *acp5b* in the transverse section of zebrafish caudal musculoskeleton tissues. WT: Tg (Ola.sp7-GFP); A: mutant of A:Tg (Ola.sp7-GFP) by crossing A mutants and Tg (Ola.sp7-GFP). The mRNA of *acp5b* was detected with Alexa Fluor 647 fluorescent probes. a: IBs and b: myoseptum.

To verify the effect of *bmp6* knockdown on osteoblast and osteoclast development, we conducted RNA-FISH of *acp5b* using Tg (Ola.sp7-GFP) zebrafish. The results of RNA-FISH showed that *sp7* was not expressed in the myoseptum of A mutants, and *acp5b* had higher expression in the myoseptum of A mutants than that in the wild-type; meanwhile, *sp7* and *acp5b* co-expressed in IBs in wild-type zebrafish (Figure 6, Supplementary Figure S9). These results indicated that after knocking out *bmp6* in zebrafish, the development of osteoblasts in the myoseptum was inhibited and the development of osteoclasts in the myoseptum was promoted.

Meanwhile, the cell cycle was influenced after *bmp6* knockout*.* Compared to the WT group, downregulated genes in group A and group M at 20 or 60 dph were enriched in hallmark E2F targets and hallmark G2M checkpoint (Figure 5). KEGG DNA replication and KEGG cell cycle were enriched by downregulated genes in the groups of 20 dph A vs. WT, 20 dph M vs. WT, and 60 dph M vs. WT (Supplementary Figure S8). The results of GSEA were similar. Hallmark E2F targets and hallmark G2M checkpoint pathways were activated in the 45 dph A group compared to the 45 dph WT group and suppressed in the 20-dph A group compared with the 20-dph WT group and the hallmark MYC targets V1, hallmark MYC targets V2, and hallmark DNA repair pathways (Figure 5).

Notably, hallmark TNFA signaling *via* the NF-KB pathway was enriched by upregulated genes at all stages in the A group compared with that in the WT group and enriched at almost all stages in the M group compared with that in the WT group except at 20 dph. Comparing A group to WT group, hallmark TNFA signaling *via* the NF-KB pathway was activated at all stages except at 45 dph in the results of GSEA. GSEA results also showed that hallmark TNF-A signaling *via* the NF-KB pathway was activated at all stages except at 20 dph in group M compared with group WT (Figure 5).

### Differential Expression Analysis of miRNA

MiRNAs with significant difference in expression (absolute log2 (fold change) > 1, *p* < 0.05) are shown in Table 1, Supplementary Figures S7C,D. Two hundred and fifteen, 84, and 24 miRNAs showed significant differential expression in caudal musculoskeletal tissues between A and WT groups at 20, 45, and 60 dph stages, respectively; while 112, 20, and 23 miRNAs showed significant differential expression in caudal musculoskeletal tissues between M and WT groups at 20, 45, and 60 dph stages, respectively. Fifty-one and 36 DE miRNAs in the muscle were obtained in the comparative groups of 60 Mdph A vs. 60 Mdph WT and 60 Mdph M vs. 60 Mdph WT, respectively.

### Pathway Enrichment Analysis of MicroRNA

miRanda and TargetScan were used to predict target genes for known miRNAs and novel miRNAs; the concordances of the two results were characterized as final target genes. A total of 10,628 target genes were predicted by miRanda, and 11,112 target genes were predicted by TargetScan, finally achieving an intersection of 10,471 target genes. The correlation coefficients of the expression level between miRNA and their target genes were calculated, and 4,372 target genes with significant negative correlation (*p* < 0.05) (Supplementary Table S13) were used as the final target genes for pathway enrichment analysis.

Pathway enrichment analysis was conducted for the target genes that were significantly negatively correlated with differentially expressed miRNA in different groups. The hallmark oxidative phosphorylation and hallmark heme metabolism pathways were enriched in the downregulated miRNA target genes in group A compared with those in group WT at all stages. Meanwhile, hallmark myogenesis and hallmark oxidative phosphorylation were enriched in the downregulated miRNA target genes in group A compared with those in M group at all stages (Figures 5E,F).

### Deficiency of *bmp6* Activated TNF-A Signaling *via* NF-KB *via sik1*

The pathway enrichment results of differentially expressed genes and GSEA both showed that TNF-A signaling *via* the NF-KB pathway is significantly activated in the *bmp6* knockout mutants at different developmental stages, and, especially it is significantly activated in the groups of 60 Mdph A and 60 Mdph M compared with 60 Mdph WT (Figure 5, Supplementary Tables S5–S9). Since tissues in 60 Mdph A, 60 Mdph M, and 60 Mdph WT had no vertebrae or spines but only muscle, myosepta, and intermuscular bones, the comparison between 60 Mdph A against 60 Mdph WT or 60 Mdph M against 60 Mdph WT could imply the influence of *bmp6* on the development of IBs, that is, TNF-A signaling *via* NF-KB might be related to IB development.

**FIGURE 7:**
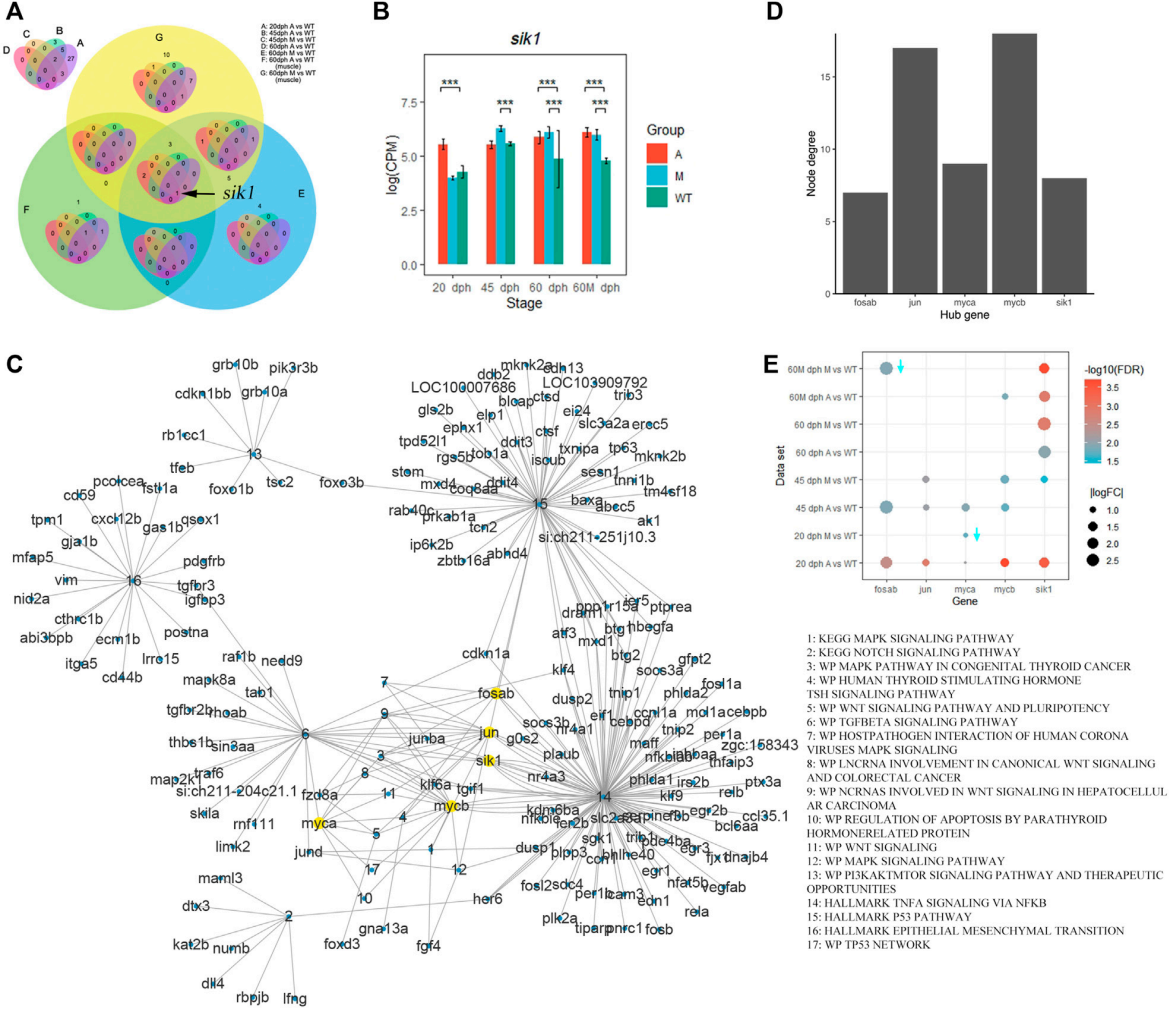
*Sik1* was a key regulatory gene of pathways related to IB development. **(A)** Venn plot of genes enriched in the TNFA signaling *via* the NF-KB pathway in different comparative groups. **(B)** Expression level of *sik1* in different groups. ***p < 0.001 **(C)** Complex gene–pathway interaction network of pathways related to bone development enriched by upregulated genes in mutants compared with wild-type. **(D)** Node degree of hub genes. **(E)** Differential expression of hub genes. Downward arrow means that logFC is less than zero.

To investigate the key regulatory genes that activated TNF-A signaling *via* the NF-KB pathway, we obtained the intersection of upregulated genes in mutants that were enriched in TNF-A signaling *via* the NF-KB pathway and found that *sik1* existed in all gene sets except in 45 dph A vs. WT (Figure 7A), and *sik1* had a higher expression level in the A group than that in the wild-type group at 20 days, 60 days, and 60 Mdph. In group M, *sik1* was also upregulated at 45 days, 60 days, and 60 Mdph compared with that in the wild-type (Figure 7B).

**FIGURE 8:**
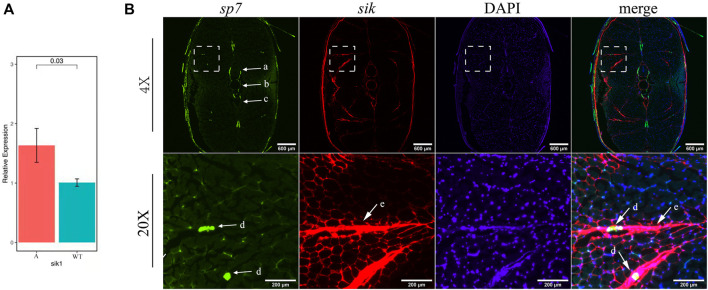
qRT-PCR and RNA-FISH of *sik1.* **(A)** Expression level of *sik1* in group A and wild-type. **(B)** *Sik1* expressed in the myospetum surrounding IBs and the mRNA of *sik1* was detected with Alexa Fluor 647 fluorescent probes. a: neural arches; b: vertebrae; c: hemal arches; d: IBs; and e: myoseptum

Furthermore, we selected pathways related to bone development from the pathways enriched by upregulated genes in mutants (Supplementary Figure S10) and established a complex gene–pathway interaction network using Cytoscape (Figure 7C). In the network, each edge connected a gene to the pathway that the gene belongs to, and node degree represented the number of pathways that a gene connected to. We found that the top five genes with the highest node degree were *mycb*, *jun*, *myca*, *sik1*, and *fosab* in this network (Figure 7D). Among these genes, *sik1* showed significantly differential expression in six datasets compared with that in the wild-type (Figure 7E). To validate this result, we performed qRT-PCR to quantify the expression level of *sik1* in groups A and WT at 90 dph. Results showed that *sik1* was significantly upregulated in group A compared with that in group WT (Figure 8A). Moreover, to analyze the spatial expression of *sik1* in zebrafish caudal tissues, we used RNA-FISH to locate *sik1* and found that *sik1* was expressed in the myospetum, and the expression of *sik1* also overlapped with *sp7* (Figure 8B). These results suggested that *sik1* might be a key regulatory gene of pathways involved in IB development and *sik1* inhibited proliferation and differentiation of osteoblasts, resulting in deletion of IBs.

## Discussion

The study of IB formation is of great significance for the bone development of fish and the aquaculture industry; it is important and difficult to identify genes related to IB formation for breeding new varieties without IBs. Nie et al. (2020) reported that knocking out *scxa* in zebrafish could reduce 70% of IBs; however, *scxa* is required for the normal development of the musculoskeleton and its knocking out caused deformed ribs (Kague et al., 2019). In this study, we obtained IB-deletion mutants by knocking out *bmp6* and found that the mutants grew normally; bone staining and micro-CT did not detect significant bone changes as well (Figure 2, Supplementary Movies S1–S3). These indicated that *bmp6* could be a key gene in the development of IBs.

In the research on the development of IBs, it has been reported that the inhibitors of TGF-β and Wnt pathways significantly delayed IB development (Nie C.-H. et al., 2019). Many signaling pathways are involved in bone regeneration, such as the Wnt/β-catenin pathway, BMP/Smad pathway, Hedgehog pathway, and Notch pathway (Yan and Alman, 2009). The key signaling pathway involved in the IB developmental stages (S1–S4) includes cyclic adenosine monophosphate (cAMP), phosphatidylinositol 3′-kinase-Akt (PI3K-Akt), mitogen-activated protein kinase (MAPK), tumor necrosis factor (TNF) signaling, and vascular endothelial growth factor (VEGF) signaling (Wan et al., 2016; Nie C.-H. et al., 2019; Li et al., 2021). P53 inhibits the expression of OSX (Sp7) to prevent bone development and osteoblast differentiation (Sinha and Zhou, 2013). TNF-α inhibits the expression of OSX and Runx2 to inhibit differentiation of osteoblasts (Barnes et al., 2003; Lu et al., 2006), and many TNF family receptors can promote osteoclast differentiation (Suda et al., 2001).

BMP6, as a member of BMPs, regulates many biological processes including iron homeostasis, fat and bone development, and reproduction. In human Sertoli cells, BMP6 promotes the proliferation and inhibits apoptosis by activating the Smad2/3/cyclin D1 pathway (Wang H. et al., 2017). Bone development can be regulated by BMP6 by activating canonical Smad-dependent pathways. Smads regulate the expression of Dlx5, Runx2, and Sp7 which are involved in osteoblast development (Chen et al., 2012). The overexpression of SP7 enhanced BMP6-induced osteoblast mineralization (Zhu et al., 2012). BMP6 also inhibits cell proliferation *via* downregulation of miR-192 (Hu et al., 2013) and inhibits stress-induced apoptosis *via* both Smad and p38 signal pathways in breast cancer cells (Du et al., 2008). Although there have been many studies on the function of the *BMP6* gene, the role of *bmp6* in the development of IBs is not clear.

**FIGURE 9:**
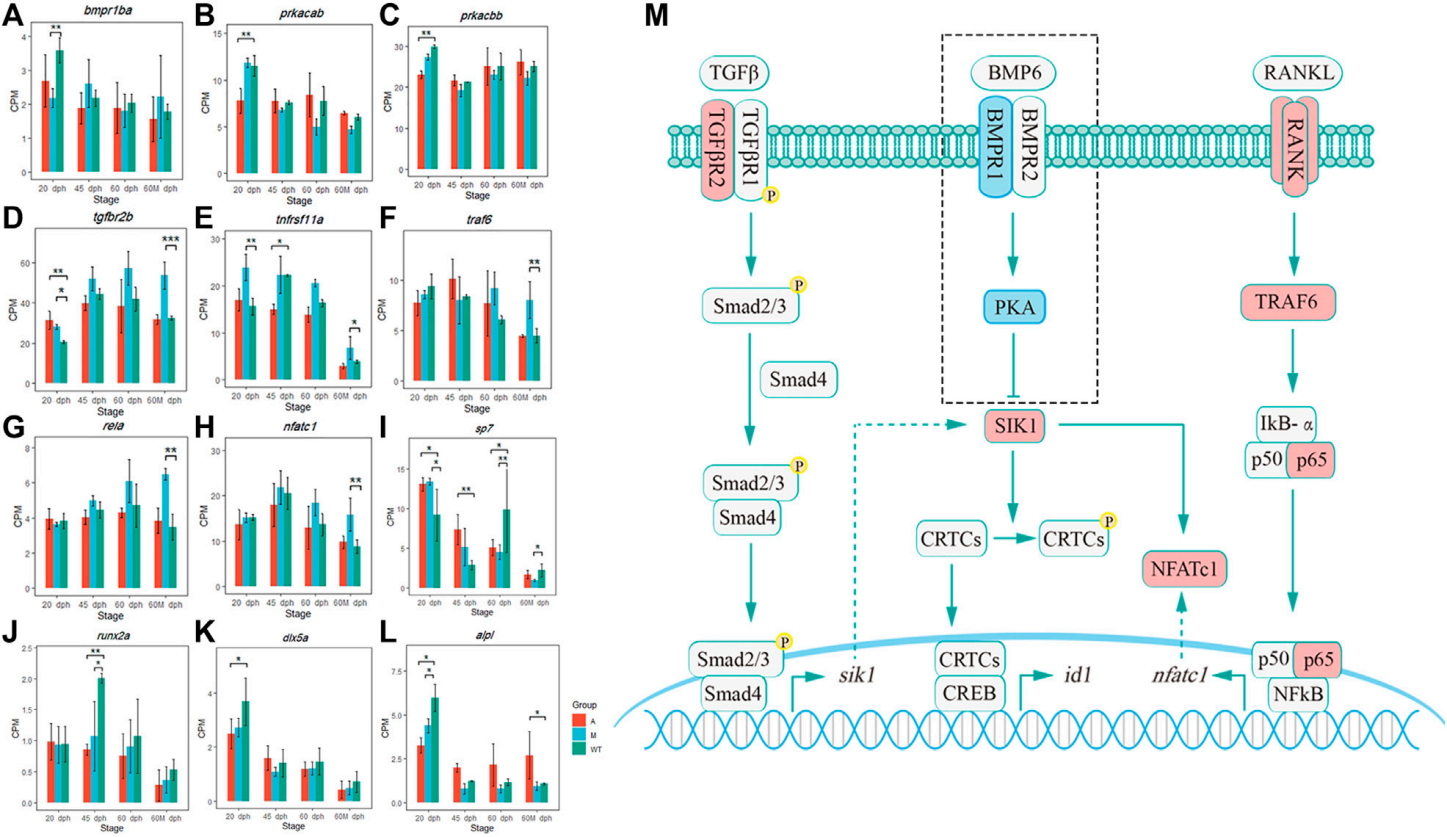
Pathway hypothesis that bmp6 regulates IB development. **(A–L)** Expression level of *bmpr1ba*, *prkacab*, *prkacbb*, *tgfbr2b*, *tnfrsf11a*, *traf6*, *rela*, *nfatc1*, *sp7*, *runx2a*, *dlx5a*, and *alpl* in different groups. *p < 0.05, **p < 0.01, and ***p < 0.001 **(M)** Pathway hypothesis that *bmp6* regulates IB development. The red box represented that its mRNA was upregulated and the blue box represented that its mRNA was downregulated in the mutants compared to that in the wild-type.

In this study, we found that the knockout of *bmp6* activated the TNF-A signaling *via* the NF-KB pathway *via* increasing the expression level of *sik1*. Previous studies showed that SIK1 is a key negative regulator of preosteoblast proliferation and osteoblast differentiation (Kim et al., 2019). BMP2 downregulates SIK1 through PKA signaling. When BMP2 signaling is absent, SIK1 phosphorylates CRTC1 and inhibits its nuclear translocation, resulting in an inhibition of CREB target genes including ID1 (Kim et al., 2019), consequently inhibiting the development of osteoblasts which was consistent with our study. In this study, the transcription of osteoblast-related genes, such as *sp7*, *dlx5a*, and *runx2a*, was influenced by the *bmp6* knockout. *Sp7* was downregulated in mutants of 60 dph and 60 Mdph; *dlx5a* was downregulated in group A compared with wild-type at 20 dph; *runx2a* was downregulated in group A and group M compared with wild-type at 45 dph; *alpl*, another marker gene of osteogenic differentiation progression, was downregulated in group A and group M compared with wild-type at 20 dph (Figures 9I–L).

SIK1 also promotes osteoclastogenesis *via* RANK/RANKL signaling, and SIK inhibitors downregulated the protein levels of NFATc1, which was a transcription factor related to the regulation of osteoclast-specific genes (Lombardi et al., 2017). In canonical NF-κB signaling induced by RANKL and TNF, RANKL and TNF recruit TRAF6 and TRAF2/5 to their receptors (RNAK and TNFR) and activate a complex consisting of IKK-α, IKK-β, and IKK-γ, leading to the release of p65/p50 heterodimers. P65/p50 translocates to the nucleus and promotes the expression of NFATc1 (Boyce et al., 2015). Moreover, the SIK gene is a direct target of the TGFβ receptor–Smad pathway. The Smad complex consisting of Smad2/3 and Smad4 binds to the enhancer of the SIK gene and promotes their expression (Lnn et al., 2012). In this study, *bmpr1ba* was downregulated in 20 dph M vs. WT (Figure 9A); *prkacab* and *prkacbb* were downregulated in 20 dph A vs. WT (Figures 9B,C); these indicated that *bmp6* knockout inhibited the expression of *pka* and BMP-PKA signaling, which further reduced the phosphorylation of SIK1 by PKA and enhanced the effect of SIK1 on downstream genes. *Tgfbr2b* was upregulated in the 20-dph A group, 20-dph M group, and 60-Mdph M group compared with that in the wild-type (Figure 9D), which suggested that TGFβ receptor signaling was activated after *bmp6* knockout, thereby promoting the expression of *sik1*. *Tnfrsf11a* (RANK) was significantly upregulated in the 20-dph M group and 60-Mdph M group (Figure 9E), and *traf6, rela* (p65), and *nfatc1* were significantly upregulated in the 60-Mdph M group compared with those in the 60-Mdph WT group (Figures 9F–H). These indicated that *bmp6* knockout promoted the development of osteoclasts by activating the NF-kB signaling pathway.

Therefore, after knocking out of *bmp6*, the expression level of *sik1* may be upregulated by the TGF-β receptor–Smad pathway and the absence of BMP-PKA signaling increased the activity of SIK1, leading to inhibition of the osteogenesis program. *Nfatc1* was upregulated through the NF-κB pathway induced by RANKL, which was necessary for osteoclast precursor differentiation. Osteoclast differentiation was promoted, and proliferation and differentiation of osteoblasts were inhibited, thereby inhibiting the formation of IBs (Figure 9M).

Bmp6 participates in the regulation of iron homeostasis (Parrow and Fleming, 2014). Bmp6 was downregulated under conditions of high iron demand (Zhang et al., 2014) and increases hepcidin expression and reduces serum iron in mice (Andriopoulos et al., 2009). The results of pathway enrichment analysis showed that the hallmark meme metabolism and hallmark myogenisis pathways (Figure 5) were enriched in upregulated genes, which were consistent with the pathway enrichment result of the target genes with significantly negative correlation to downregulated miRNAs (Figure 5). Hallmark heme metabolism consists of genes involved in the metabolism of heme (a cofactor consisting of iron and porphyrin) and erythroblast differentiation. In zebrafish, miRNA-30a regulates myogenesis by downregulating Sik1 (Jenean et al., 2014). Our results showed that the biological mechanism of iron metabolism and development of skeletal muscle may be related to some novel miRNAs, which were affected by *bmp6*.

## Conclusion

In this study, we obtained an IB-lacked mutated zebrafish strain and an IB partial deletion mutated zebrafish strain by knocking out different sites in *bmp6* and found that *bmp6* played an important role in IB formation. Deficiency of *bmp6* inhibited osteoblast development and promoted osteoclast differentiation in the myoseptum. Knockout of *bmp6* inhibited IB development by activating TNF-A signaling *via* NF-KB and increasing *sik1* expression level.

## Data Availability

The datasets presented in this study can be found in online repositories. The names of the repository/repositories and accession number(s) can be found below: https://ngdc.cncb.ac.cn/gsa, CRA004567.
