# Supplementary material for: Transcriptomic Analysis Provides Insights to Reveal the bmp6 Function Related to the Development of Intermuscular Bones in Zebrafish
Source: Front Cell Dev Biol. 2022 May 12;10:821471. doi: 10.3389/fcell.2022.821471 (PMC9135397; doi:10.3389/fcell.2022.821471)
Supplement: Supplementary file 5 [file Image1.PDF]

Supplementary Material

Transcriptomic analysis provides insights to reveal the *bmp6* function related to development of intermuscular bones in zebrafish

Huan Xu[1,2,3,7†], Guangxiang Tong[1,2,3,†], Ting Yan[1,2,3†], Le Dong[1,4], Xiaoxing Yang[1,4], Dongyu Dou[1,4], Zhipeng Sun[1,2,3], Tianqi Liu[1,2,3], Xianhu Zheng[1,2,3], Jian Yang[5], Xiaowen Sun[1,2,3],Yi Zhou[6], Youyi Kuang[1,2,3,*]

Affiliations

1.Heilongjiang River Fisheries Research Institute of Chinese Academy of Fishery Sciences, Harbin , China.

2.National and Local Joint Engineering Laboratory for Freshwater Fish Breeding, Harbin , China.

3.Key Laboratory of Freshwater Aquatic Biotechnology and Breeding, Ministry of Agriculture and Rural Affairs, Harbin , China.

4. College of Fisheries and Life Science, Shanghai Ocean University, Shanghai , China.

5. Institute of Mariculture Breeding and Seed Industry, Zhejiang Wanli University, Ningbo , China

6. Stem Cell Program of Boston Children's Hospital, Division of Hematology/Oncology, Boston Children's Hospital and Dana Farber Cancer Institute, Harvard Medical School, Boston, MA , USA.

7. Heilongjiang Provincial Key Laboratory of Hard Tissue Development and Regeneration, The Second Affiliated Hospital of Harbin Medical University, Harbin 150086, China

[†]These authors contributed equally to this work.

* To whom correspondence should be addressed. Youyi Kuang, E-Mail: kuangyouyi@hrfri.ac.cn

Contents

Supplementary Figure 1 Schematic of sample separation in group 60Mdph

Supplementary Figure 2 RNA-FISH of *bmp6* and co-expression analysis of *sp7*, *tnmd*, *scxa*, *xirp2a* in transverse section of zebrafish caudal musculoskeleton tissues.

Supplementary Figure 3 Bone staining of head and fin skeletons of the mutants and the wild-type zebrafish at 60dph.

Supplementary Figure 4 Quality control assessment of RNA sequenced reads

Supplementary Figure 5. Distribution and saturation of junctions

Supplementary Figure 6 Principal component analysis (PCA) and Pearson correlation coefficients of all samples

Supplementary Figure 7 Differentially expressed genes and miRNAs

Supplementary Figure 8 KEGG and GO pathways enriched by DEGs and GSEA

Supplementary Figure 9 RNA-FISH of asp5b using Tg(Ola.sp7-GFP) zebrafish.

Supplementary Figure 10 Bone-related pathways from KEGG, WikiPathways and Hallmark database enriched by up-regulated genes

Supplementary Table S1. Sequences of probes used for RNA-FISH and primers used for qRT-PCR.

Supplementary Table S2. Statistical results of alignment of mRNA reads

Supplementary Table S3. Statistical results of alignment of miRNA reads

Supplementary Table S4. Body weight and body length data of mutants and wild type at 72hpf and 90dph

Supplementary Table S5. Hallmark Pathways enriched by DEGs

Supplementary Table S6. KEGG pathways enriched by DEGs

Supplementary Table S7. WIKI Pathways enriched by DEGs

Supplementary Table S8. Biological Processing GO terms enriched by DEGs

Supplementary Table S9. Enriched Hallmark pathways analyzed by GSEA

Supplementary Table S10. Enriched KEGG pathways analyzed by GSEA

Supplementary Table S11. Enriched WIKI pathways analyzed by GSEA

Supplementary Table S12. Enriched biological processing GO terms analyzed by GSEA

Supplementary Table S13. DE miRNAs and their target genes with significant negative correlation

1 Supplementary Figures

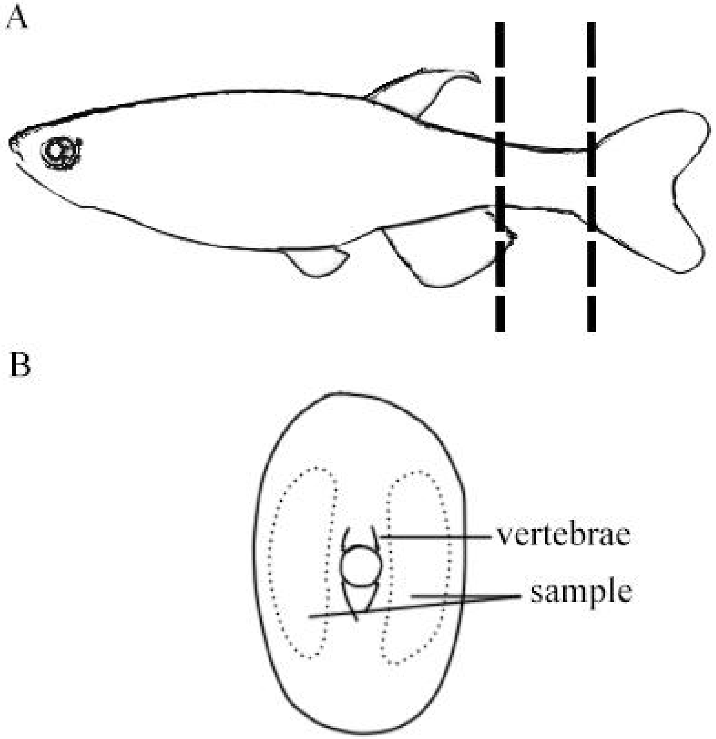

Supplementary Figure 1 Schematic of sample separation in group 60Mdph. (A) The caudal musculoskeletal tissues (between the posterior of anal fin and the anterior of caudal fin) were collected. (B) The muscles on either side of the vertebrae were collected.

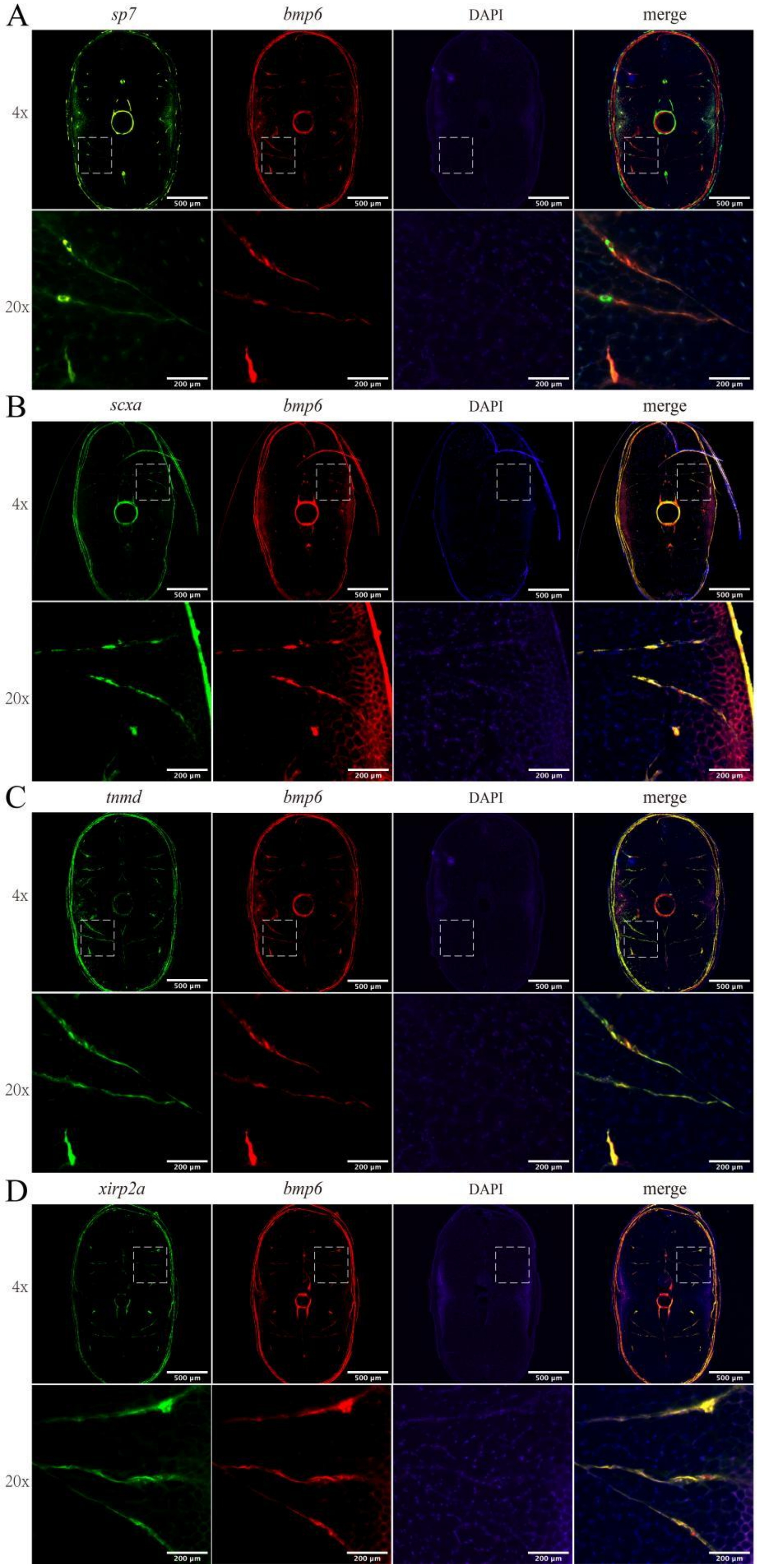

Supplementary Figure 2 RNA-FISH of *bmp6* and co-expression analysis of *sp7*, *tnmd*, *scxa*, *xirp2a* in transverse section of zebrafish caudal musculoskeleton tissues. Intermuscular bones were shown in dotted line boxes and were observed with 20×.

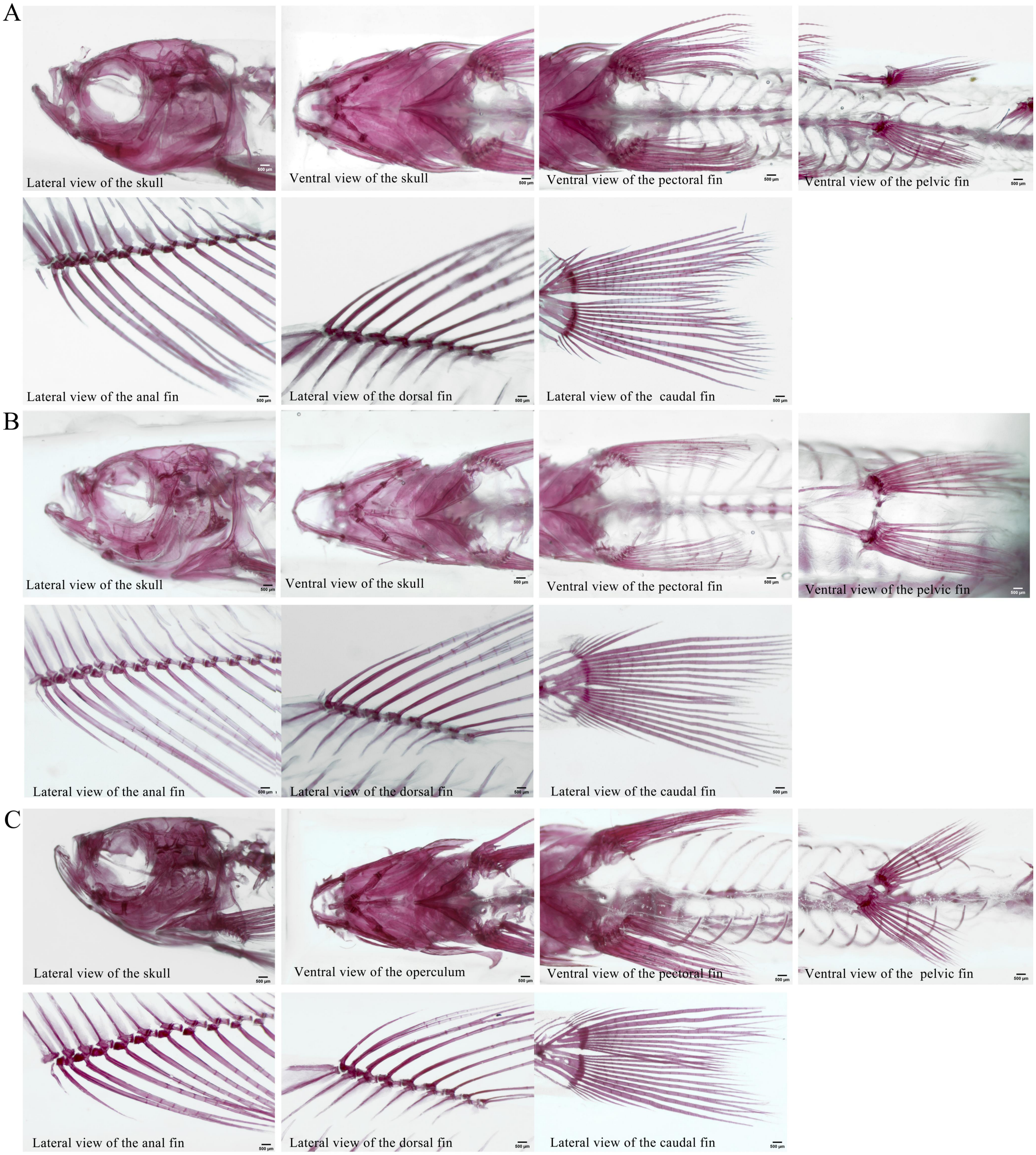

Supplementary Figure 3 Bone staining of head and fin skeletons of the mutants and the wild-type zebrafish at 60dph. Bone staining of the A group (A), the M group (B), and the wild type (C).

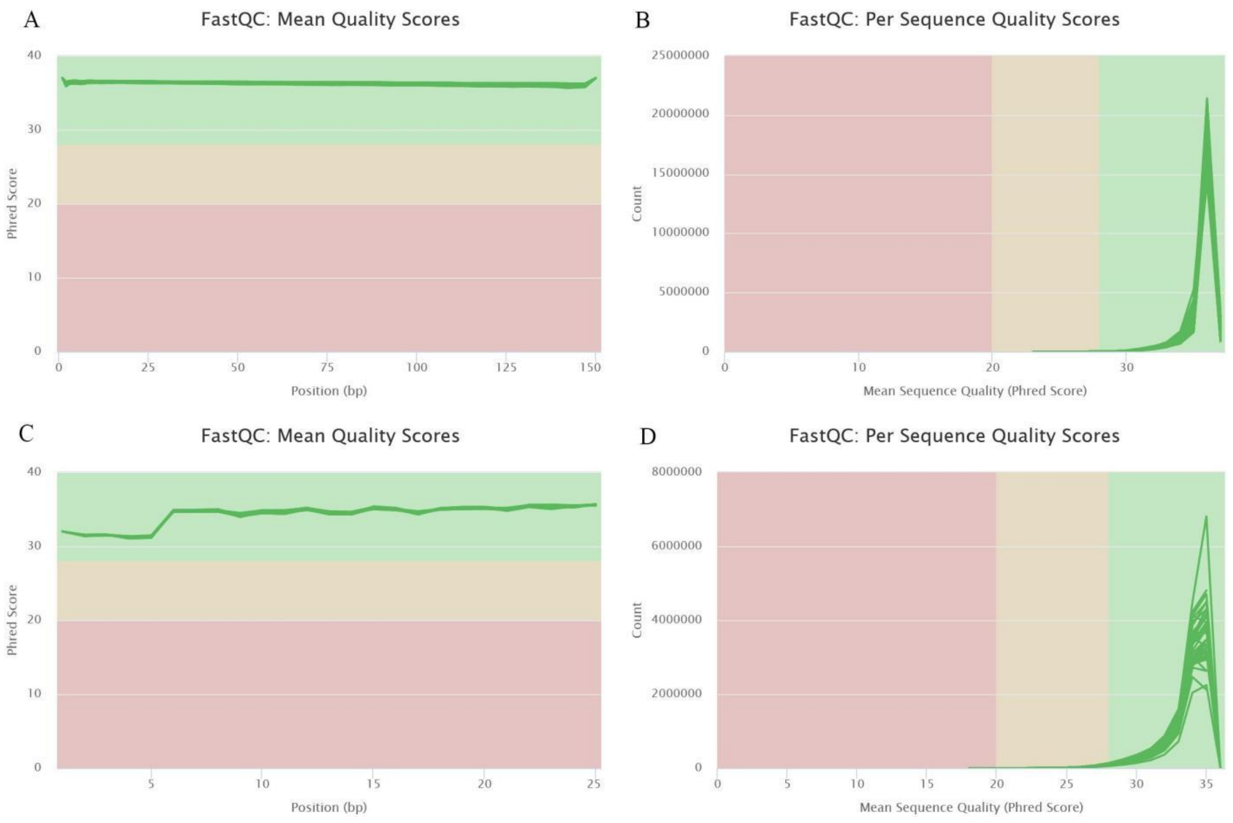

Supplementary Figure 4 Quality control assessment of RNA sequenced reads. (A) Mean quality scores per read of trimmed reads from mRNA sequencing in 37 samples. (B) Mean quality scores per position of trimmed reads from mRNA sequencing in 37samples. (C) Mean quality scores per read of trimmed reads from miRNA sequencing in 37samples. (D) Mean quality scores per position of trimmed reads from miRNA sequencing in 37samples.

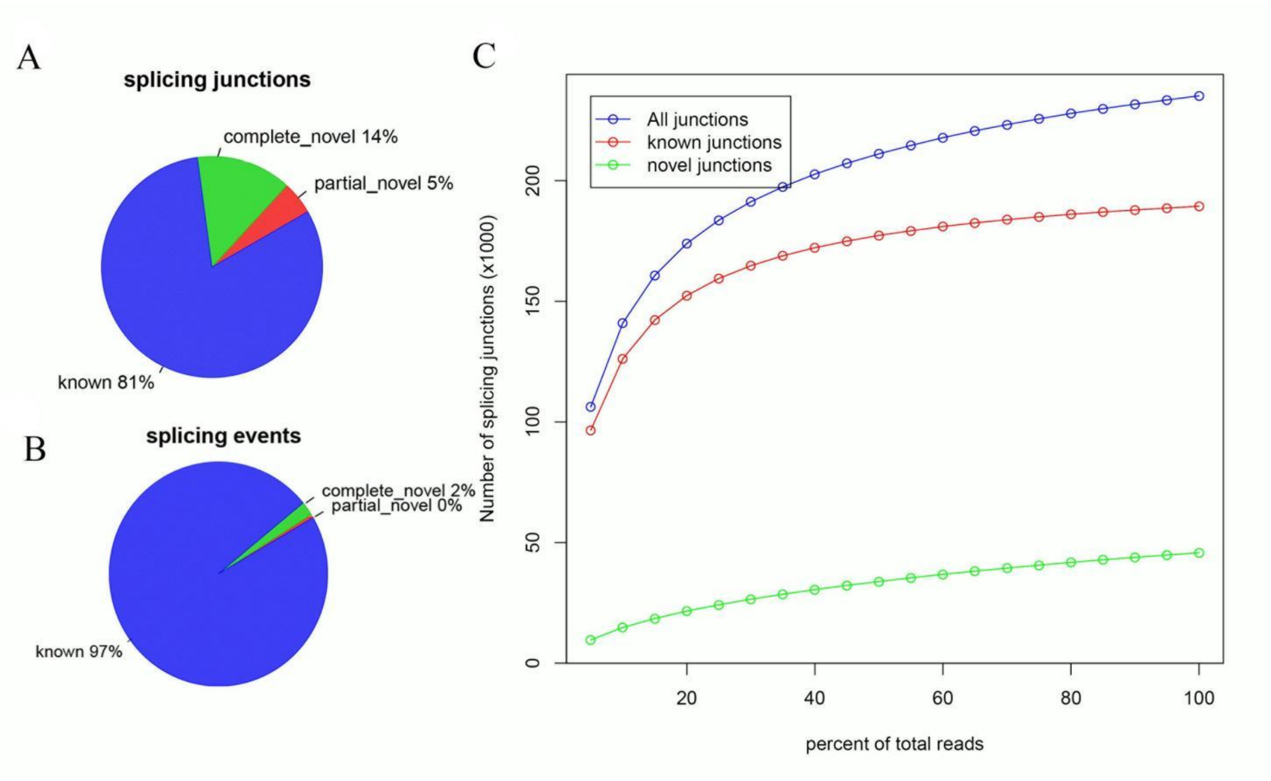

Supplementary Figure 5 Distribution and saturation of junctions. This figure was drawn using data from sample B20A2. (A) Splicing annotation in splice junction level. Known: The junction is part of the gene model with both splice sites, 5' splice site (5'SS) and 3'splice site (3'SS) are annotated by reference gene model. Complete_novel: Both 5'SS and 3'SS are novel. Partial_novel: One of the splice sites (5'SS or 3'SS) is novel, and the other splice site is annotated. (B) Splicing annotation in splice event level. (C) Saturation of junctions. X-axis represents percent of resampling of total reads, y-axis represents detected splice junctions and compared to reference gene model from each subset.

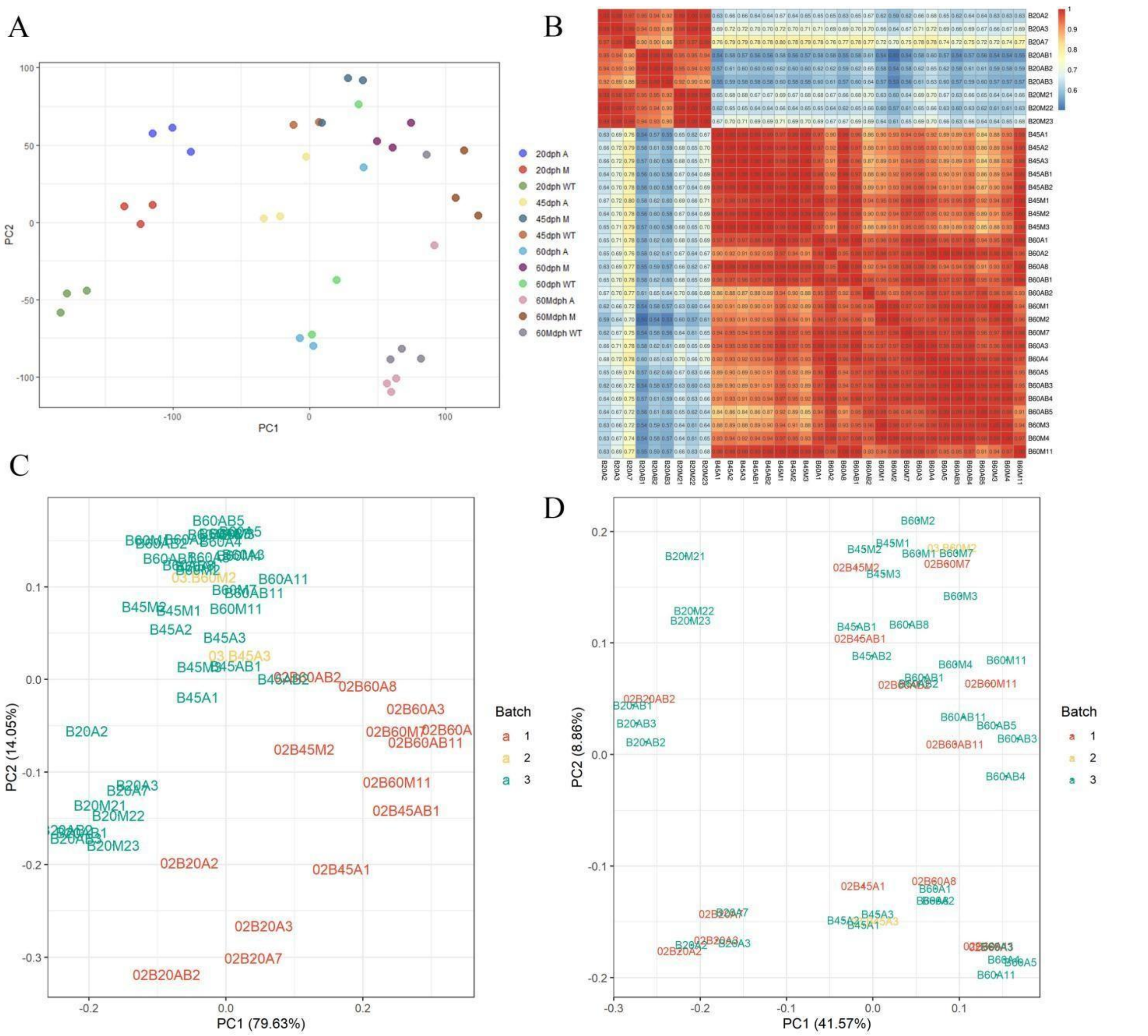

Supplementary Figure 6 Principal component analysis (PCA) and Pearson correlation coefficients of all samples. (A) PCA of mRNAs' normalized CPM expression values. (B) Pearson correlation coefficients of mRNAs' expression data of all samples. (C) PCA of miRNAs' normalized CPM expression values before removing batch effects. (D) PCA of miRNA expression level after removing batch effects.

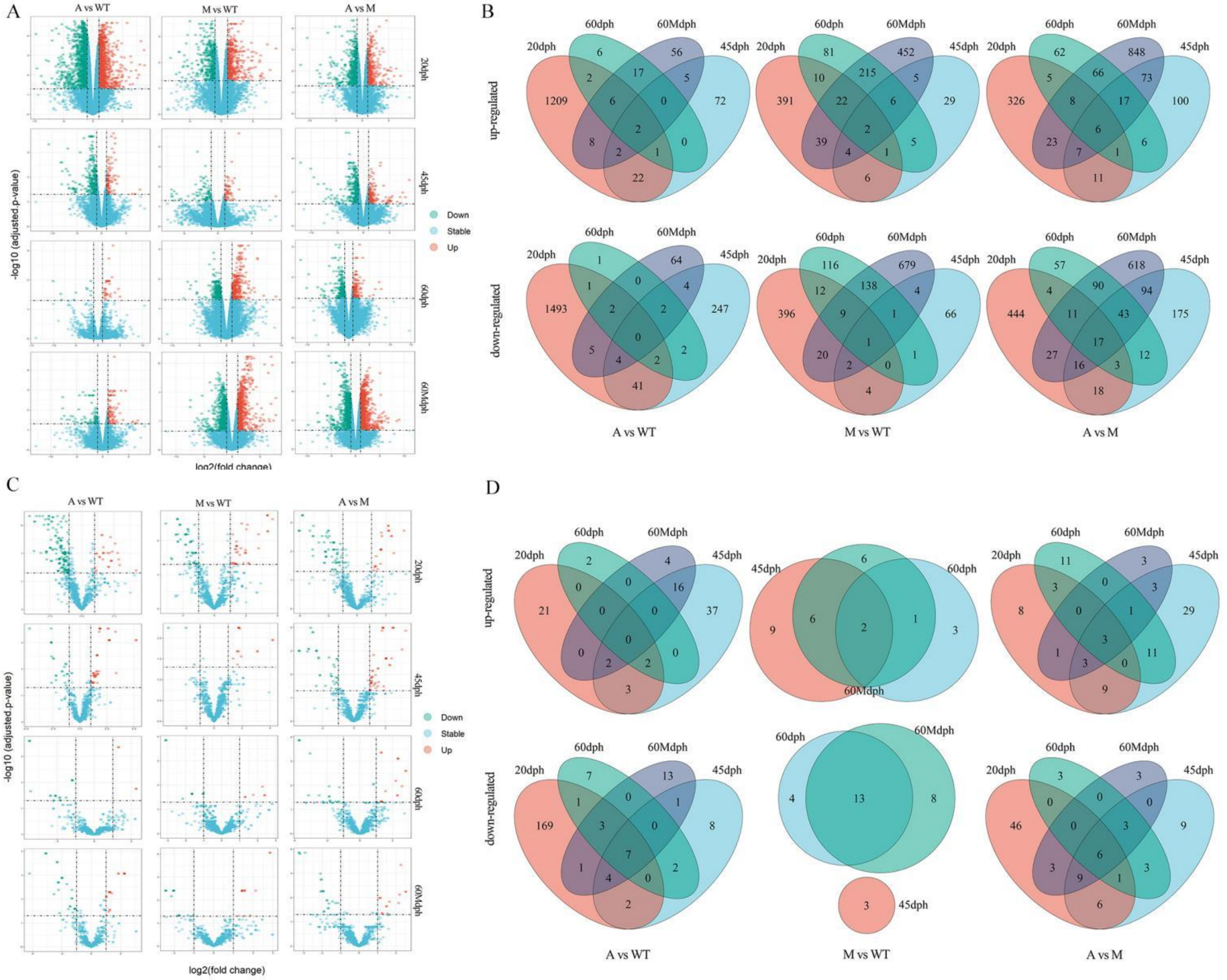

Supplementary Figure 7 Differentially expressed genes and miRNAs. (A) Volcano maps of DEGs between groups. Red bubbles represent up-regulated mRNAs and green bubbles represent down-regulated mRNAs. (B) Venn plots of DEGs between groups. (C) Volcano maps of DE miRNAs between groups. (D) Venn plots of DE miRNAs between groups.

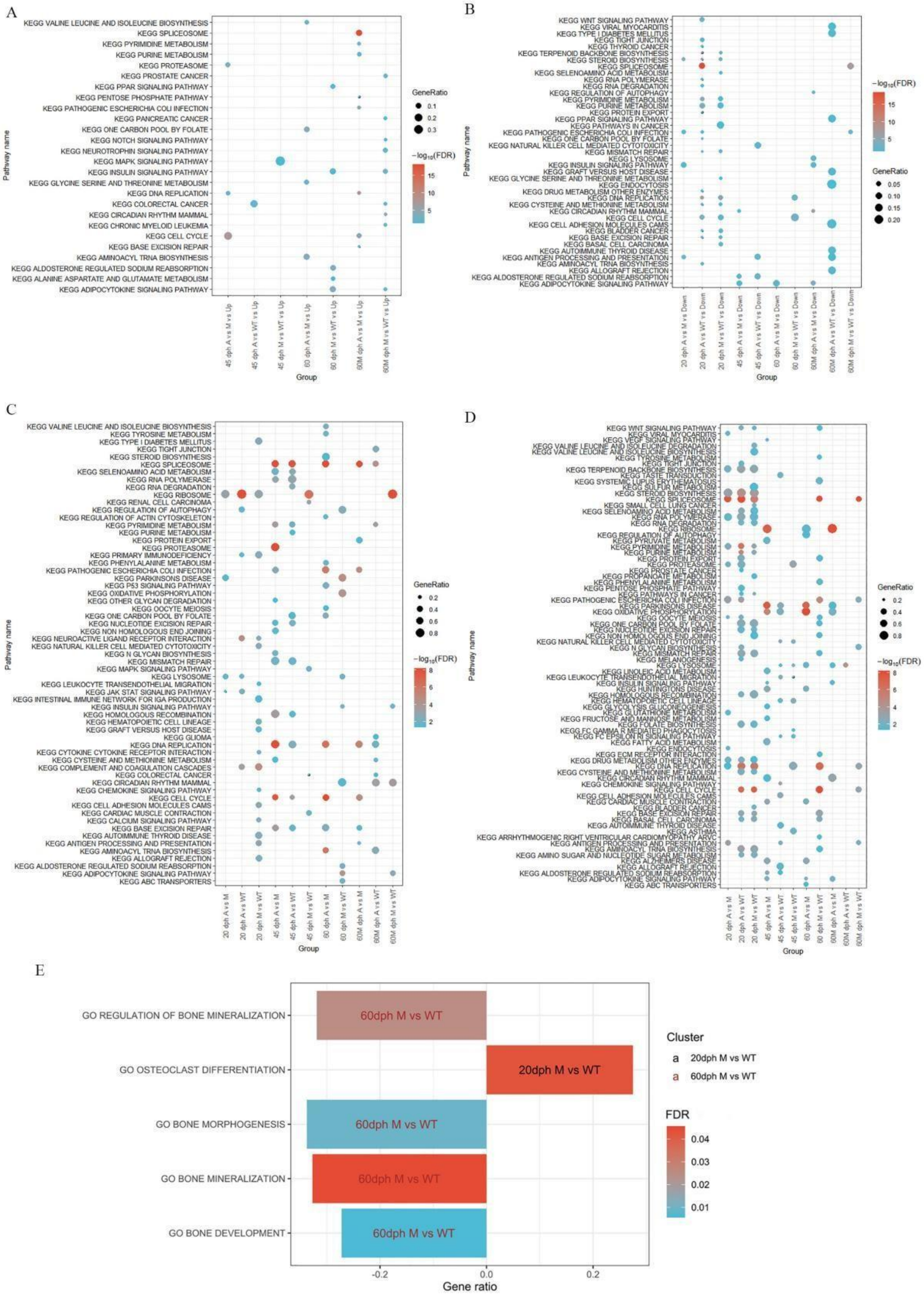

Supplementary Figure 8 KEGG and GO pathways enriched by DEGs and GSEA. (A) KEGG pathways enriched by up-regulated DEGs. (B) KEGG pathways enriched by down-regulated DEGs. (C) Activated KEGG pathways analyzed by GSEA. (D) Suppressed KEGG pathways analyzed by GSEA. (E) GO pathways related to bone development enriched by DEGs.

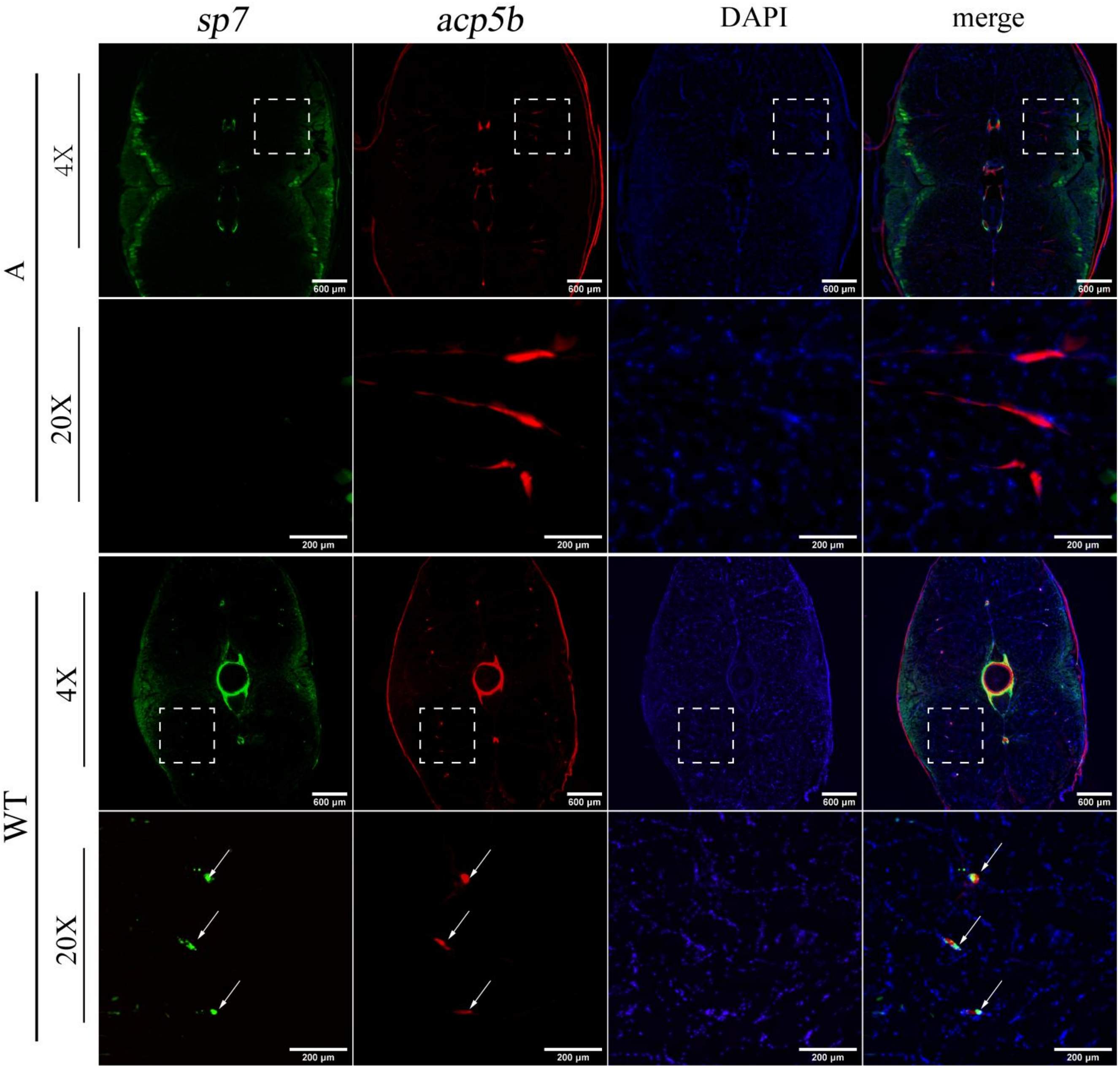

Supplementary Figure 9 RNA-FISH of *asp5b* using Tg(Ola.sp7-GFP) zebrafish. Intermuscular bones in wild type zebrafish were shown in dotted line boxes and were observed with 20×, marked with arrows.

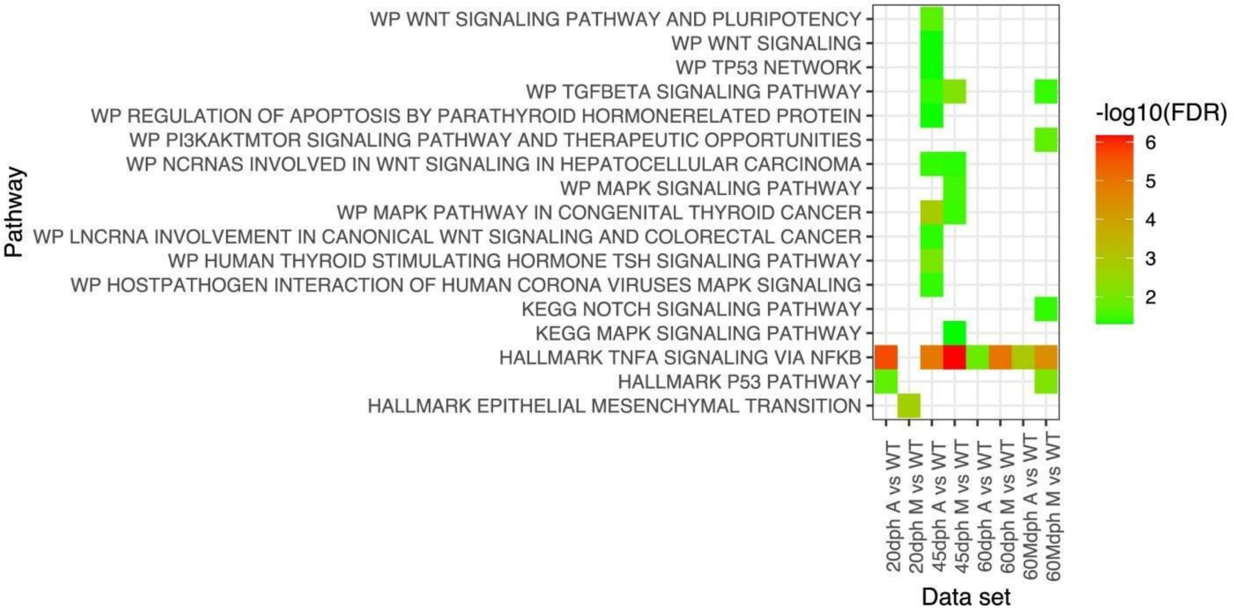

Supplementary Figure 10 Bone-related pathways from KEGG, WikiPathways, and Hallmark database enriched by up-regulated genes

2 Supplementary Tables were included as a separate Excel file.
